# Predictive decision making driven by multiple time-linked reward representations in the anterior cingulate cortex

**DOI:** 10.1038/ncomms12327

**Published:** 2016-08-01

**Authors:** Marco K. Wittmann, Nils Kolling, Rei Akaishi, Bolton K. H. Chau, Joshua W. Brown, Natalie Nelissen, Matthew F. S. Rushworth

**Affiliations:** 1 Department of Experimental Psychology, University of Oxford, South Parks Road, Oxford OX1 3UD, UK; 2 Department of Brain and Cognitive Sciences, Center for Visual Science, University of Rochester, Rochester, New York 14627, USA; 3 Department of Rehabilitation Sciences, The Hong Kong Polytechnic University, Hong Kong, China; 4 Department of Psychological and Brain Sciences, Indiana University, Bloomington, Indiana 47405, USA; 5 Centre for Functional MRI of the Brain, University of Oxford, John Radcliffe Hospital, Oxford OX3 9DU, UK

## Abstract

In many natural environments the value of a choice gradually gets better or worse as circumstances change. Discerning such trends makes predicting future choice values possible. We show that humans track such trends by comparing estimates of recent and past reward rates, which they are able to hold simultaneously in the dorsal anterior cingulate cortex (dACC). Comparison of recent and past reward rates with positive and negative decision weights is reflected by opposing dACC signals indexing these quantities. The relative strengths of time-linked reward representations in dACC predict whether subjects persist in their current behaviour or switch to an alternative. Computationally, trend-guided choice can be modelled by using a reinforcement-learning mechanism that computes a longer-term estimate (or expectation) of prediction errors. Using such a model, we find a relative predominance of expected prediction errors in dACC, instantaneous prediction errors in the ventral striatum and choice signals in the ventromedial prefrontal cortex.

## Abstract (web summary)

Past experiences and future predictions both shape our decisions. Here, the authors trained participants in a foraging task in which reward rates varied systematically over time and find the dACC tracks both recent and past reward rates, leading to opposing effects on decisions about whether to stay or leave a reward environment.

## Graphical abstract

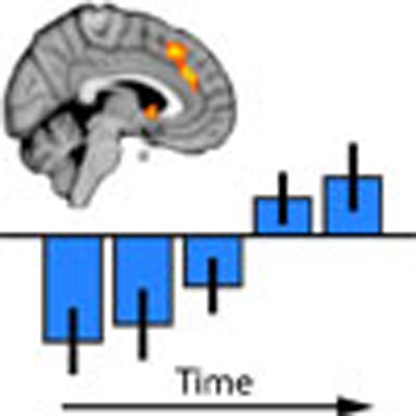

Many decisions are based on past experience because we expect the values of choices to be stable or to change only gradually. To choose effectively, we must either track these values or, ideally, anticipate the future value of a choice. While much is known about how choice values are learned from past experience, it is relatively unknown how people predict in advance not only that a value will change but also how it will change. This problem lies at the heart of many judgment tasks, such as stock market trading or behavioural adaptation to the continuously changing reward rates animals and humans experience in foraging patches^1^,^2^. A simple way, however, to predict future choice values is to estimate the change in rewards. This can be performed by comparing recent and past reward rates.

Evidence comparison is central to decision making^3^ and value comparison is central to reward-guided decision making. Ventromedial prefrontal cortex (vmPFC) signals reflect the comparison of choice values when making decisions^4^. VmPFC blood oxygen-level-dependent (BOLD) activity scales with the value difference between available options, and the chosen and unchosen options have dissociable positive and negative effects^4^,^5^,^6^,^7^, which can be understood as reflecting a value competition process^8^,^9^,^10^,^11^. Previous studies focused on decisions between two clearly defined and concrete options usually associated with specific stimuli. While this is clearly an important decision-making mode, behaviour often also reflects alternation between continuous engagement in the same behaviour (for example, when an animal repeatedly forages in one patch or a person maintains engagement with the same task) with behavioural change (when the animal moves to an alternative patch or the person switches task)^1^,^2^,^12^,^13^. In such situations it is critical to know prospectively how profitable the next repetition of an action will be. For some time there has been evidence that evaluation of a choice reflects comparison against previous choice values. For instance, rats' approach speed to a given reference amount of food pellets depends on their previous reward experience. Having experienced more rewards previously, rats are slower to approach the reference amount of food pellets; contrarily, having experienced fewer rewards beforehand, they are quicker to approach the same amount of food. These phenomena are known as positive and negative successive contrast effects, respectively^14^,^15^. Similarly, deciding whether to further commit to, or to quit, engagement in an action, the critical comparison is not between two stimulus values but between elements of the past reward history.

Brain mechanisms comparing recent and past reward rates require fine-grained information about the past history of rewards. Dorsal anterior cingulate cortex (dACC) is necessary for retaining the history of encountered rewards^16^,^17^, and neurophysiological studies have demonstrated dissociable influences of past and recent rewards on the activity of dACC neurons^18^,^19^. In addition, activity in the dopaminergic system and striatum also reflects reward history^20^,^21^,^22^. Reward history signals here have been understood in the context of reinforcement-learning models^23^ that involve computation of a reward prediction error (PE) relative to previous outcomes^24^. However, typically, simple reinforcement learners cannot weight recent and past rewards in an opposing manner. To do this, a learning mechanism requires a contrast mechanism comparing recent and past reward rates^15^,^25^,^26^. We used functional magnetic resonance imaging (fMRI) in humans to investigate value comparisons of recent and past reward rates. We find that dACC holds multiple time-linked reward representations simultaneously, predictive of the way past and recent rewards guide decisions to stay or to leave an environment. Our results suggest a key role for dACC in computation of reward trajectories and the transformation of decision variables to choice.

## Results

### Experimental design

**Figure 1:**
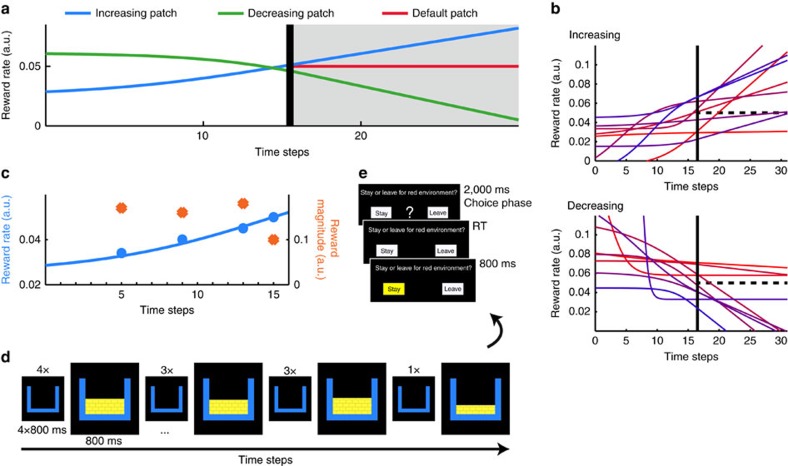
Experimental design and implementation. (**a**) Two example patches with increasing (blue) and decreasing (green) reward rates. At LSDs (black) subjects chose between staying in the patch and switching to a default patch with a stable reward rate (red). In these examples, correct decisions (stay on blue, leave green) can be predicted from the reward rate curves. (**b**) All 18 reward rate curves from which trials were derived. Solid black line indicates LSD and vertical dashed line indicates reward rate of reference patch. For visualization purposes only, different colouring for reward rate curves was used, and the curves were aligned so that the LSD is on the same time step. (**c**) Sequence of events corresponding to the blue reward rate curve before LSD in **a**. Four reward events were presented at time steps 5, 9, 13 and 15. Their reward rates (blue dots), which conform to the reward rate curve (blue line), are calculated by dividing their reward magnitudes (orange dots) by the time delay from the previous reward event or the start of the patch. (**d**) Screen during events in **c**. Empty boxes represent non-reward events; the height of ‘gold bars' in reward events represent their reward magnitudes. Each event was displayed for 800 ms. Between events, a fixation cross was shown and subjects proceeded to the next event by pressing a button. Note that lastRR for this patch would be equivalent to the height of the gold bars in the final box divided by two time steps. (**e**) LSD followed, without time jitter, after the last reward event (boxes in the default environment were red; therefore, it was labelled ‘red environment' for subjects).

We designed a reward-learning task in which subjects chose to further commit to, or to leave, a foraging-like patch based on its estimated future value. The patches were characterized by reward rate trends that could be discerned by comparing past and recent reward rates. If the decision maker knows the reward rates at two different time points in the past, they have sufficient information to judge whether the reward rate has increased or decreased between these time points. In environments with monotonic reward rate changes such knowledge can be exploited to extrapolate the reward trend and predict the future value of the patch. Patches were derived from reward rate curves similar to those in optimal foraging theory^2^ (Fig. 1a,b and Supplementary Fig. 1). The patches consisted of sequences of time steps on which either reward or non-reward events occurred (Fig. 1c,d). The reward events were spread out such that their reward rates conformed to the underlying reward rate curves. Subjects proceeded from time step to time step by pressing a button. At a predetermined time step, subjects were offered a leave–stay decision (LSD; Fig. 1e). For LSDs, subjects had to consider the 15 further time steps they would encounter after LSD, and decide, for this time period, whether to stay and further explore the environment they were in or to leave and re-engage with a pre-learned default environment with a stable reward rate. LSDs should be based on a comparison of the anticipated value of the current environment (the sum of rewards that would be delivered after LSD) and the pre-learned value of the default environment.

### Opposing effects of recent and past rewards on choice

**Figure 2:**
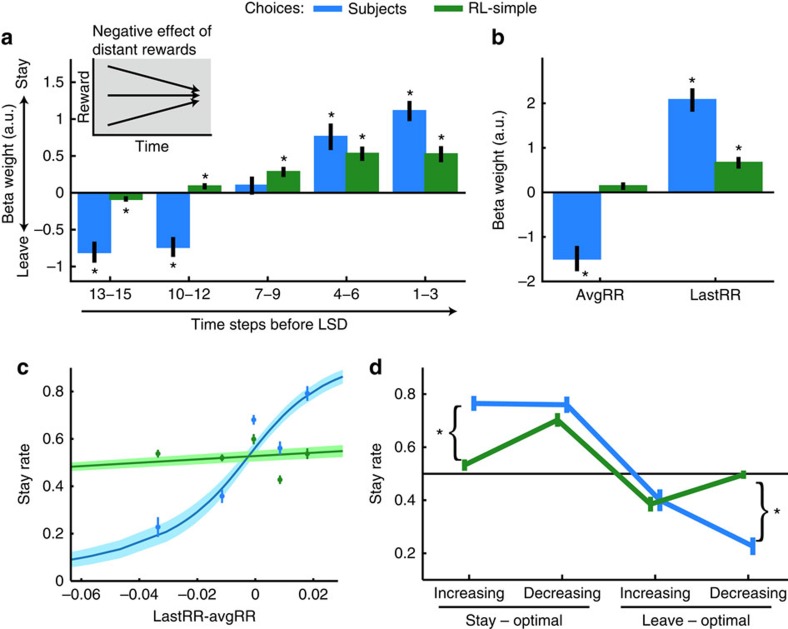
Recent and past reward rates influence choice in an opposing manner. (**a**) GLM predicting stay choices. Binning the reward history into bins of three time steps relative to the LSD (LSD-1-3, LSD-4-6 and so on) revealed the influence of discrete previous time steps on choice. Opposing effects of recent and past rewards emerged gradually in subjects' behaviour (blue). A simple-RL model (green) captured positive effects of recent rewards but failed to represent distant rewards as negatively as subjects did. Inset: the reason for the negative impact of early rewards becomes particularly clear when keeping recent rewards stable. The reward trajectory points more upwards the lower its starting position. (**b**) GLM predicting stay choices. As in **a,** reward rate trend-guided behaviour can be explained by a positive effect of a patch's most recent value (lastRR) in combination with a negative effect of reward rates in the past (avgRR). RL-predicted choices (green) captured part of the positive influence of lastRR on human subjects (blue), but failed to represent avgRR negatively. (**c**) Softmax functions of subjects' actual (blue) and RL-simple predicted (green) stay rates plotted against lastRR–avgRR illustrates that, overall, subjects' choices were influenced by the reward rate change in contrast to RL-simple. Overlaid are binned actual and RL-predicted stay rates. (**d**) Stay rates plotted by optimal choice and categorical reward rate trend. The simple-RL model's choices (green) were close to random when the reward rate trend was predictive of the optimal choice (stay/increasing and leave/decreasing). It performed similar to subjects (blue) when the reward rate trend had to be ignored. (*P<0.0001; error bars are s.e.m. between subjects).

We measured the influence of rewards occurring at different times during a trial on the LSD using a general linear model (GLM). Therefore, we divided the reward history into reward rate bins reflecting reward received over five sets of three time steps each moving backwards in time from the LSD (LSD-1-3, LSD-4-6, and so on; Fig. 2a). In our experiment, reward rates in these different time bins share less than 25% of their variance; therefore, we can estimate their influences on choice behaviour. Note also that this means that we could also test whether choices were solely based on a patch's initial reward rates or whether later reward rates had an additional influence on choice (correlation between initial and last time bin: *r*=0.02), which turned out to be the case. While subjects tended to stay in a patch when reward rates in recent bins were high (LSD-1-3: *t*_19_=10.12; *P*=4 × 10^−9^; LSD-4-6: *t*_19_=4.98; *P*=8 × 10^−5^), the more distant the reward rate bins were the more negative the effect of high reward rates on the decision to stay (LSD-10-12: *t*_19_=−6.85; *P*=2 × 10^−6^; LSD-13-15: *t*_19_=−6.99; *P*=10^−6^).

We compared subjects' choices with choices made by an individually fitted simple Rescorla–Wagner reinforcement-learning (RL-simple) model^23^. We used the value estimate of the model at the time of the LSD (that is, after observation of the last reward outcome) as decision variable. We applied the former GLM to RL-simple-simulated choices (Fig. 2a, green bars). RL-simple captures recent positive effects of rewards (LSD-1-3: *t*_19_=6.44; *P*=4 × 10^−6^; LSD-4-6: *t*_19_=7.58; *P*=4 × 10^−7^), but is unable to simulate a negative influence of past reward rate bins on choices to the same degree as seen in human subjects (paired *t*-tests on last two bins: LSD-10-12: *t*_19_=8.11 *P*=1 × 10^−7^; LSD-13-15: *t*_19_=6; *P*=9 × 10^−4^). The strongly negative effects of rewards in past time bins in our human subjects (LSD-10-12, LSD-13-15) resemble successive contrast effects in animals^14^ (inset Fig. 2a).

The temporal gradient in Fig. 2a can be summarized using two parameters: the reward rate of the last reward event (lastRR) before the LSD and the average reward rate (avgRR) throughout the whole period before the LSD (Fig. 2b). These two regressors capture the negative and positive effects of past and recent rewards on human behaviour (lastRR: *t*_19_=9.56; *P*=10^−7^; avgRR: *t*_19_=−6.23; *P*=5 × 10^−6^). While the RL-simple model explained the lastRR effect (*t*_19_=7.78; *P*=3 × 10^−7^), it falsely predicted a positive instead of a negative effect for avgRR (*t*_19_=3.53; *P*=0.0023; see Supplementary Fig. 2a,b for a description of the complementarity of Fig. 2a,b). The difference between lastRR and avgRR (lastRR−avgRR) gives a measure of reward rate trend, accounting for both increasing (positive) and decreasing (negative) reward rate trends (Fig. 2c and Supplementary Fig. 2c,d). As our investigations focused on the use of any type of monotonic reward trend in choice, lastRR−avgRR assumes the simplest type of trend, a linear one, rather than, for example, an exponential trend. Note, however, that subjects based their choices not only on lastRR−avgRR, but also on the absolute size of lastRR (Supplementary Fig. 2c).

The shortcomings of RL-simple become particularly clear when binning the trials by their categorical reward rate trend (increasing or decreasing) and sorting them by optimal choices (defined by maximal payoff; Fig. 2d). When choices had to be made against the reward rate trend (decreasing/stay-optimal and increasing/leave-optimal), RL-simple's choice predictions were close to the choices observed in subjects (although for decreasing/stay-optimal trials, we found a small but significant difference *t*_19_=2.44; *P*=0.025). In these cases, correct choice could be based on lastRR alone. However, when optimal decisions depended on the reward rate trend (increasing/stay-optimal and decreasing/leave-optimal), human behaviour was strikingly more optimal than that predicted by RL-simple (*t*_19_=9.72; *P*=8 × 10^−9^ and *t*_19_=−10.31; *P*=3 × 10^−9^, respectively). These results corroborate earlier results (Supplementary Fig. 2c) that subjects base their choices on both the instantaneous reward rate at the time of choice and the reward rate trend.

In sum, we show that subjects' choices were influenced by recent and past reward rates in an opposing manner. Because a simple-RL model integrates historical and recent rewards into a single estimate of an option's value, it does not represent recent and historical rewards with opposing weights and, therefore, does not make choices like the human subjects.

### Estimating PEs enables trend-guided choice

Prediction errors (PEs; differences between the reward outcomes that choices actually led to and prior reward expectations) are a simple measure of the option's reward rate change. Because PEs have a role in value-updating they have been linked to learning^23^; however, it is also possible that PEs may be used as a decision variable to guide decisions^25^. We tested whether an RL model that computes an expectation of PEs is able to model reward trend-guided choice.

We modified a simple-RL model based on a type of average reward rate model that has recently been described^25^. The computation of the average reward rate offers an estimate of the reward opportunities of an environment^26^. Often the average reward rate reflects the opportunities that are currently foregone by engaging in one particular choice, a proxy for the value of leaving a patch^27^. However, in many natural environments choice values change over time, for example, the choice to forage from a tree becomes less valuable as the tree depletes. In such a setting, the average reward rate does not reflect the value of many concurrently alternative options, but instead reflects the longer-term average value of the currently exploited option. Comparison of the option's recent value against its longer-term average value^15^ produces a simple measure of reward rate change—from an RL perspective, the PE.

**Figure 3:**
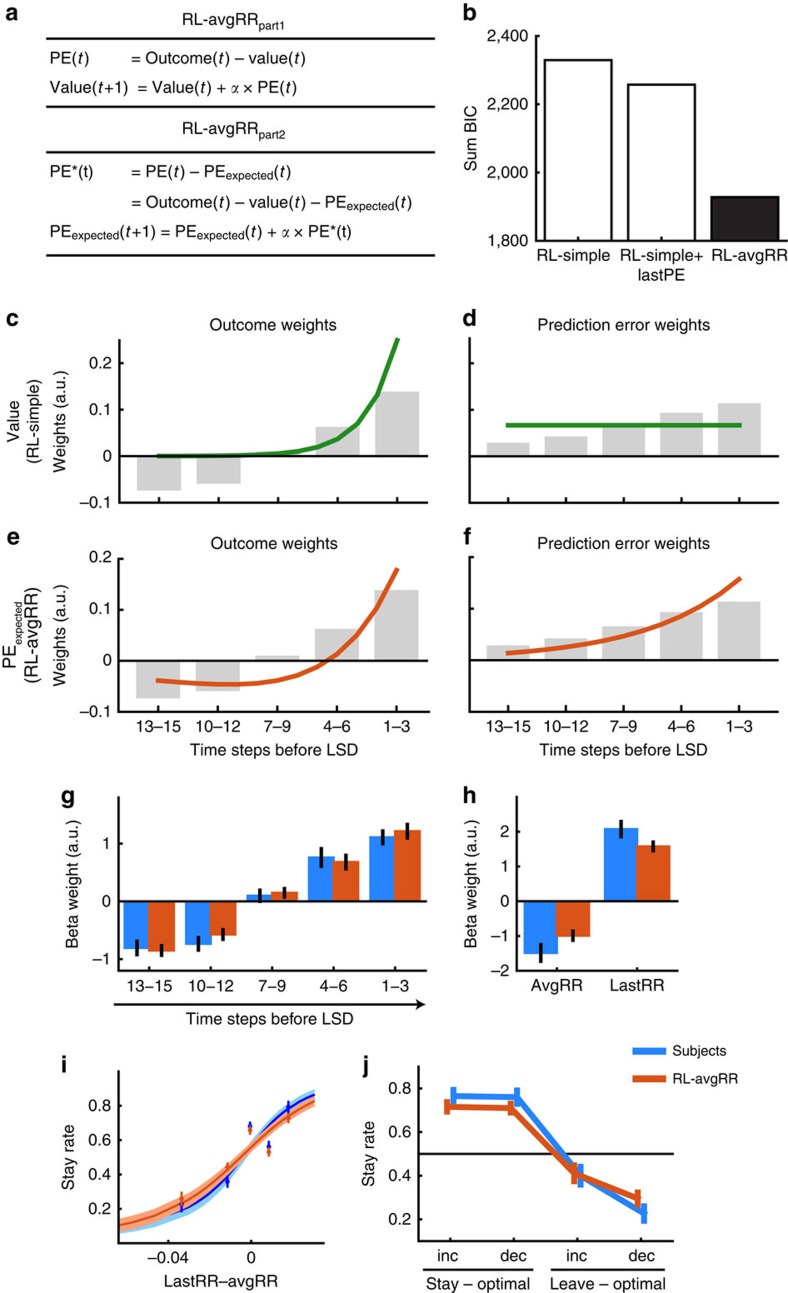
RL-avgRR explains reward rate trend-guided choices. (**a**) Equations used in RL-avgRR. (**b**) Summed Bayesian information criterion (BIC) scores for RL-avgRR were lower than RL-simple and RL-simple+lastPE, indicating better model fit. (**c**–**f**) Comparison of past outcome and PE weights used by a standard value estimate and PE_expected_ at the time of choice (same *x*-axis in all plots). Grey bars indicate the weights of influence that past outcomes (**c**,**e**, same data) and past PEs (**d**,**f**, same data) had on subjects' decision to stay in a patch. The lines indicate the amount of influence past events had on the calculation of a simple value estimate (from RL-simple; green line) and PE_expected_ (from RL-avgRR; orange line). Note that the empirically determined weights correspond qualitatively to the weights used by RL-avgRR, but not to RL-simple. While simple value estimates are a recency-weighted sum of past outcomes (**c**), expected PEs are highest when encountering high rewards after initially poor outcomes (negative than positive weighting, **e**). The same information as in **c**,**e** can be presented as a function of PEs for RL-simple (**d**) and RL-avgRR (**f**). Again, the influence of past PEs on subjects' choices are qualitatively similar to the way PE_expected_ is calculated from past PEs. (**g**–**j**) Analyses from Fig. 2 were repeated for RL-avgRR. Unlike RL-simple (Fig. 2), RL-avgRR made choice predictions (orange) that were similar to subjects' actual choices (blue). Note, in particular, how the weights in 3 g mimic the theoretical weights shown in **e** and that RL-avgRR is able to represent past rewards negatively. See Fig. 2 for legends.

The modified model, RL-avgRR, consisted of two hierarchically organized RL-learning mechanisms (Fig. 3a). The first one (RL-avgRR_part1_; Fig. 3a, top) was a standard RL model (as RL-simple) and as such computed (standard) PEs on every trial. The second learning mechanism, RL-avgRR_part2_ (Fig. 3a, bottom) used an estimate of PEs, PE_expected_, which was updated on every time step using a higher-order PE, PE* (that is, the difference of observed PE and expected PE). Over time, PE_expected_ learned a recency-weighted estimate past PEs. PE_expected_ at the time of the LSD was used as a decision variable.

We compared the goodness of fit of the RL-avgRR model with RL-simple. Moreover, we tested whether there is evidence that subjects' indeed used a longer-term estimate of PEs rather than only a single, most recent PE. For this, we considered a more sophisticated version of RL-simple that uses, in addition to RL-simple's last value estimate, RL-simple's last PE to make a choice (RL-simple+lastPE; Methods). To investigate the goodness of fit, we calculated the Bayesian information criterion, which penalizes additional free parameters. RL-avgRR was, by far, the best of the three models (Fig. 3b and Supplementary Fig. 3a; lower Bayesian information criterion values indicate better model fit). We also examined a range of alternative and related models (Supplementary Note 1). For instance, we tested whether different learning rates for RL-avgRR_part1_ and RL-avgRR_part2_ would improve the model fit. While such a modification might be useful in other cases it was not in our case. RL-avgRR proved to be the best fitting simple model (Supplementary Fig. 3b,c). In particular, the inclusion of RL-avgRR_part1_'s value estimate directly in the decision variable did not further improve model fit, although it may be useful in other settings (Supplementary Fig. 4).

Computing expected PEs implies a very specific weighting of past outcomes and PEs. This weighting, that is, the way events at different times in the past influence expected PEs, is consistent with the weighting of past events expressed in subjects' choices. It might be best explained in comparison with simple value estimates. For instance, given by the delta rule^23^, RL-simple's value estimate can be calculated as the recency-weighted sum of past reward outcomes (Fig. 3c, green line). The weighting means that the value estimate varies more as a function of recent rewards than as a function of longer-term past rewards, but all rewards have a positive weight of influence. This weighting profile is inconsistent with our finding that longer-term past outcomes have a negative influence on subjects' tendency to stay in a patch (grey bars in Fig. 3c, also blue bars in Fig. 2a). PE_expected_, however, weights recent rewards positively and past rewards negatively (Fig. 3e, orange line) similar to the weights expressed in subjects' choices; just as PEs are highest when encountering high rewards after experiencing low rewards beforehand^24^. The function expressed by the orange line is given by the learning rate and the model's equations alone. It is independent of other free parameters used in the model (Supplementary Fig. 3b,c).

Expected PEs and simple value estimates can be decomposed into the weights of influence of past outcomes (Fig. 3c,e). They can, alternatively, also be decomposed into the weights of past PEs (Fig. 3d,f). Also, from this perspective, the weighting function of PE_expected_ is qualitatively similar to the weights expressed in subjects' choices. The computation of expected PEs for choice, therefore, offers a simple explanation of the positive and negative time-linked reward effects that characterize reward trend-guided choice.

Applying RL-avgRR to our experimental schedule simulates the type of behaviour we observed in subjects. We repeated the four analyses we applied to RL-simple in Fig. 2 using RL-avgRR instead. Just as seen in actual human behaviour, RL-avgRR predicted a smooth transition in the negative-to-positive effects of more distant versus more recent rewards on choices (Fig. 3g). Analogously, this can be summarized as simultaneous positive and negative effects of lastRR and avgRR (Fig. 3h). Consequently, RL-avgRR predicted choices in accordance with the reward rate trend (Fig. 3i) and also choices in cases where it was optimal to follow the reward trend as well as the recent reward rate (Fig. 3j). Overall, these analyses show that, in contrast to RL-simple (Fig. 2), choice values produced by RL-avgRR are negatively influenced by longer-term past rewards. This enabled the model to extrapolate reward trends.

In sum, a RL-based learning mechanism that tracks not only the longer-term history of encountered rewards, but also the longer-term history of PEs, can be used to model reward trend-guided choice.

### dACC contrasts recent and past rewards

**Figure 4:**
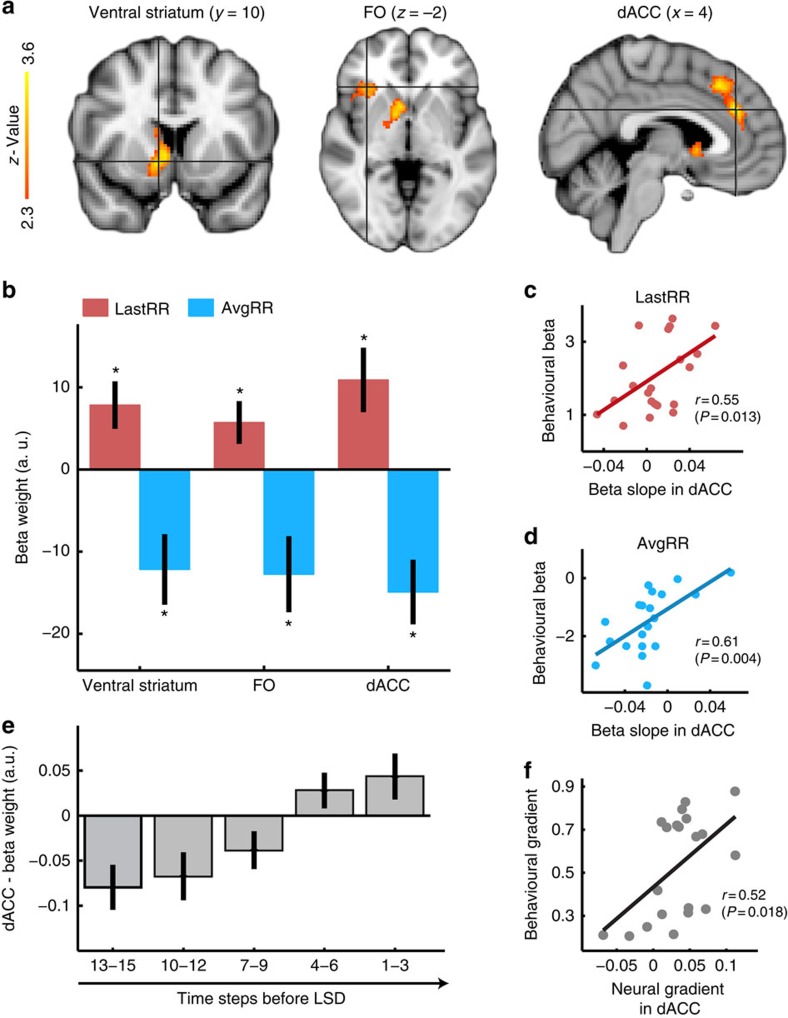
Opposing effects of recent and past reward rates in dACC predict choice. (**a**) A whole-brain contrast lastRR−avgRR time-locked to the LSD revealed three areas in which a more positive reward rate trend led to more activity: dorsal anterior cingulate cortex (dACC), right frontal operculum (FO) and right ventral striatum. (Family-wise error cluster-corrected, *z*>2.3, *P*<0.05). (**b**) ROI analyses of the three areas (using leave-one-out procedures) show separate neural responses to lastRR and avgRR. For all areas, extracted beta weights indicate that lastRR had a positive effect, while avgRR had a negative effect. (**c**,**d**) In dACC, the slopes of the behavioural beta weights for both lastRR (**c**) and avgRR (**d**) were predictive of how much the respective recent and past reward rates influenced subjects' choices. Neither of the other areas showed either correlation. (**e**) In dACC, we validated the results found in **b** by analysing the neural effects of reward rates presented in discrete time bins before the LSDs (analogous to the behavioural analysis in Fig. 2a). We found a temporal gradient of reward effects on BOLD activity that was similar to the temporal gradient of reward effects on behaviour in Fig. 2. (**f**) Using this alternative analysis approach, we were again able to confirm a relationship between dACC activity and behaviour; in dACC, but in neither of the other areas, the gradient of neural responses to past rewards was predictive of the behavioural gradient (Fig. 2a) characterizing the influence of past rewards on the decision to stay or leave (error bars are s.e.m.; *P<0.05).

We began our fMRI analysis by identifying brain regions involved in the computation of the reward rate trend using a parametric approach that does not rely on an RL model. We used the regressor lastRR−avgRR, which reflects the reward rate trend. Activity in the dACC, right ventromedial striatum and right frontal operculum (FO) increased at the time of the LSD when the reward rate trend was more positive, leading subjects to stay in an environment (Fig. 4a and Supplementary Table 2). We went on to conduct regions of interest (ROIs) analyses using a leave-one-out procedure to identify individual ROIs (Supplementary Methods). All ROI analyses were time-locked to the LSD.

To investigate whether reward rate trend effects in dACC were, as hypothesized, composed of opposing signals indexing recent and past reward rates, we obtained separate estimates of activity related to lastRR and avgRR in the same GLM. In dACC (Fig. 4b), beta weights indicated a positive effect of lastRR (*t*_19_=2.78; *P*=0.012) and a negative effect of avgRR (*t*_19_=−3.8; *P*=0.001), recalling the positive and negative effects of these variables on behaviour (Fig. 2b). To examine the relationship between individual differences in neural effects and behavioural effects, we estimated slopes of signal increase as indices of evidence accumulation^9^,^28^ in dACC. As previously, we used slopes of signal increase because they are simply and intuitively related to evidence accumulation processes that may occur in dACC^12^,^28^, although it is possible that other aspects of dACC BOLD are also related to behaviour. The dACC avgRR slope in each subject correlated with the weight each subject placed on the same information when making a decision (*r*=0.61; *P*=0.004; Fig. 4d). A similar relationship between dACC lastRR slope and lastRR behavioural weight confirmed the relationship (*r*=0.55; *P*=0.013; Fig. 4c). Similar analyses revealed that, although lastRR and avgRR were associated with opposing effects in FO and ventral striatum (all *t*_19_>|2.2|; all *P*<0.041), there were no correlations between neural and behavioural effects (all *P*>0.05). Additional control analyses showed that lastRR−avgRR was not confounded by response selection difficulty (Supplementary Fig. 5a). Moreover, inclusion of response selection difficulty^29^ in a GLM did not change the lastRR−avgRR signal in dACC (Supplementary Fig. 5b). This is consistent with previous findings that foraging-related decision variables were encoded in dACC beyond an unspecific effect of task difficulty in this brain region^28^,^29^,^30^.

To validate our first analysis, we used a complementary approach to investigate the reward effects in dACC in a temporally more detailed manner. We binned rewards in five time steps as in our behavioural analysis (Fig. 2a). This allowed investigation of the parametric effects of rewards encountered at multiple time points in the past on present BOLD activity. We extracted the group peak signals for each time bin using a time-course analysis (Supplementary Methods). For dACC neural results were, again, strikingly similar to behavioural results (Fig. 4e, compared with Fig. 2a), a gradient from positive to negative effect sizes was observed as reward rates became more distant in time (see Supplementary Fig. 6a,b for FO and ventral striatum results). In the behavioural analysis the gradient in beta weights reflects how rapidly the impact of reward rates on the choice to stay in a patch reversed with time. In dACC, but not in FO or the ventral striatum, the neural gradient predicted the behavioural choice gradient (*r*=0.52; *P*=0.018; Fig. 4f; see Supplementary Fig. 6 for details on the gradient calculation and for additional control analyses).

In sum, regardless of the precise manner in which BOLD signals were decomposed, dACC signals predicted the positive effects of recent and the negative effects of past rewards on choices. The opposing time-linked signals observed do not suggest that dACC and the other regions integrate rewards to a simple mean estimate (as RL-simple would), but instead point towards a comparison of recent and past reward rates necessary for the computation of reward trends.

### PEs and choice in medial prefrontal cortex

We next examined the neural mechanisms of reward trend-guided choice based on the reinforcement-learning model that captured subjects' choices best: RL-avgRR. For each subject, we derived trial-by-trial parameters from an individually fitted RL-avgRR model. First, we used the decision variable of RL-avgRR, the expected PE at the time of the LSD, in a whole-brain GLM time-locked to the LSD. The expected PE allows subjects to make inferences about the future rewards after the LSD. We again identified an activation cluster in dACC (Supplementary Fig. 7a) and also the right inferior frontal junction^31^.

We went on to investigate the relationship between expected PEs, choice and instantaneously experienced PEs in two analyses. Both analyses were time-locked to the LSD and used the value of the expected PE at the time step of the last reward delivery, that is, one time step before LSD, not yet updated by the last reward event: PE_expected_(LSD−1). For simplicity, we refer to this parameter just as PE_expected_.

**Figure 5:**
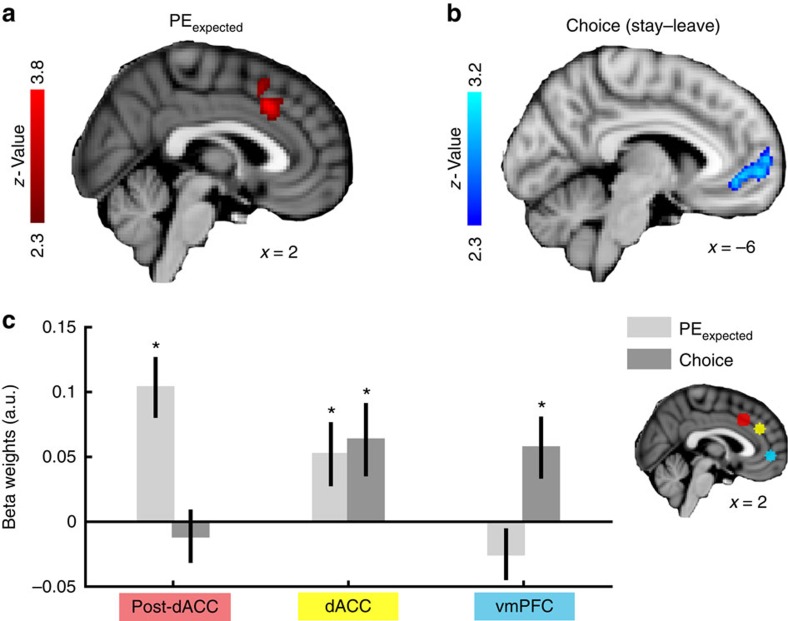
Separable representations of choice and decision variables along the ACC. (**a**) At the time of choice, activity in a posterior region of the dACC (post-dACC) varied as a function of PE_expected_. Activity was largely confined to the anterior rostral cingulate zone. The same was the case previously for dACC activity related to the reward rate trend (Fig. 4a). (**b**) The vmPFC BOLD signal increased when subjects decided to stay in a patch compared with leaving it. (**c**) Analysis of BOLD response to the model-based evidence for staying in a patch (PE_expected_) and the ensuing choice along an axis of post-dACC (red, from Fig. 5a), a more anterior dACC region (yellow, lastRR−avgRR contrast from Fig. 4a) and vmPFC (blue, from Fig. 5b). Anterior dACC signals encode both PE_expected_ and the ensuing choice to commit to or leave the patch. Contrarily, post-dACC and vmPFC show only a significant effect of PE_expected_ and the categorical choice, respectively. Note that the anterior dACC ROI was identified using a leave-one-out procedure. Significance of PE_expected_ in post-dACC and choice in vmPFC was assessed in the previous whole-brain analysis (Fig. 5a,b). (*P<0.05, one-sample *t*-test; error bars are s.e.m. between subjects).

In a first GLM, we aimed to dissociate neural representations of the reward trend-related decision variables from the decisions they drive. We used a binary choice regressor and PE_expected_, which were sufficiently decorrelated so that both could be included in the same GLM (Supplementary Fig. 7b). We found strong effects of PE_expected_ in several brain regions (Supplementary Table 2) including dACC (Fig. 5a) but excluding the FO or ventral striatum. By contrast, vmPFC was more active for stay compared with leave choices (Fig. 5b) consistent with reports that vmPFC activity reflects choice computations in a variety of contexts^5^,^8^,^32^,^33^,^34^. We examined these effects in detail by placing ROIs over the vmPFC and dACC peak coordinates (*posterior dACC* in Fig. 5c) as well as over the closely adjacent dACC peak we identified in the first fMRI analysis in Fig. 4 (*dACC* in Fig. 5c). Note that both ACC ROIs were within the anterior rostral cingulate zone^35^ and that we used a leave-one-out procedure to select the *dACC* ROI. We also used a leave-one-out procedure to avoid temporal bias in the signal analysis (Supplementary Methods). Results suggested that vmPFC only carried information about the ultimate stay/leave decision and not about the expected PEs on which the decision was based, while *posterior dACC* only carried information about the expected PEs but not the ultimate stay/decision decision. *dACC*, however, exhibited significant effects of both expected PEs (*t*_19_=2.26; *P*=0.037) and choice (*t*_19_=2.37; *P*=0.029; Fig. 5c). This replicates our previous result (Fig. 4), suggesting the presence of both reward and choice-related effects in *dACC*. To consolidate our results, we repeated this analysis and added a task difficulty regressor^29^ (as calculated above; Methods). As in our previous control analysis (Supplementary Fig. 5b) reward signals and choice signals in *dACC* remained significant (Supplementary Fig. 7c).

In a second GLM, we aimed to identify neural correlates of the component parts of RL-avgRR. As regressors, we used the three component variables that the model needs to track at the time point before choice (*t*=LSD−1): PE_expected_, value and outcome. Value and outcome refer to the model's simple value estimate and to the magnitude of the last reward event, respectively (see Fig. 3a). In particular, we investigated whether brain regions encoded a standard PE, that is, increased their activity as a function of outcome, but decreased their activity as a function of value (contrast outcome minus value)^36^. Note that, for this analysis, RL-avgRR was fitted on all subjects to ensure that, for all subjects, the three regressors of interest shared less than 25% of their variance.

**Figure 6:**
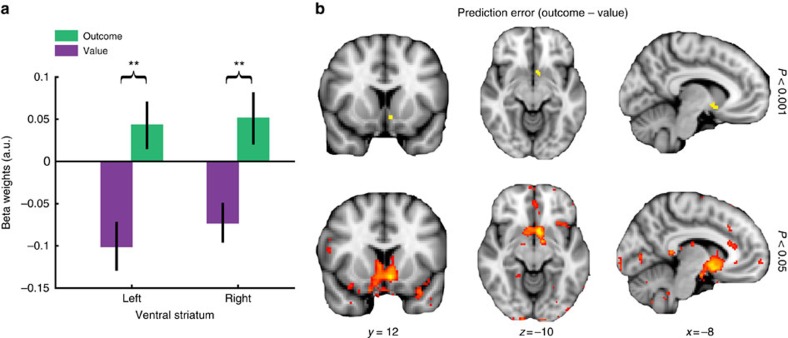
Standard PEs in the ventral striatum. (**a**) Significant standard PE (outcome minus value) signals in the left and right ventral striatum. ROIs are the same as for the striatal ROI in Fig. 4b as well as the same ROI mirrored to the contralateral side. For each subject and both hemispheres, ROIs were determined via a leave-one-out procedure to avoid spatial bias. (**b**) Whole-brain PE contrast shown at two thresholds (shown for illustration only). The PE signal is centred on the left ventral striatum (top row; threshold of 0.001, uncorrected). No other brain region showed an equally strong encoding of standard PEs (bottom row; threshold of 0.05, uncorrected). Note that images are shown according to the radiological convention, so left/right is flipped. *x*/*y*/*z* coordinates refer to both images from both rows. (**P<0.01, paired *t*-test; error bars are s.e.m. between subjects).

Again, PE_expected_ identified a cluster in dACC, among other regions (Supplementary Table 2). On the basis of previous reports^37^, we strongly expected the ventral striatum to encode standard reward PEs. To test this, we used the ventral striatal ROI from our previous analyses (Fig. 4b), which was derived using a leave-one-out procedure, as well as the same striatal ROI mirrored to the contralateral side (individually for each subject to avoid bias). Indeed, we found significant PE signals (contrast outcome minus value) bilaterally in the ventral striatum (left: *t*_19_=3.31; *P*=0.004; right: *t*_19_=3.04; *P*=0.007; Fig. 6a). This PE effect did not survive standard whole-brain cluster correction. However, when lowering the cluster-forming threshold (|*z*|>2.3, *P*<0.15), the first PE signal to reach whole-brain significance was centred on the left ventral striatum (signal peak at −6/12/−10 in Montreal Neurological Institute (MNI) atlas; Fig. 6b).

In sum, we found that dACC (and not FO or the ventral striatum) most reliably encoded the expected PE across our analyses. Moreover, the ventral striatum was most sensitive to instantaneous standard PEs, and vmPFC showed signs of choice-related activity in the absence of a specific representation of the reinforcement history.

## Discussion

Considerable emphasis has been placed on the way both human and animal behaviour is guided by value expectations based on learned associations between specific stimuli and reward outcomes. In many natural-foraging situations, however, part of what animals are doing may simply be deciding whether to continue a behaviour or whether it will be more profitable to change behaviour^1^,^12^. This is particularly important in environments, in which no immediate cues act as instructions to stop or switch behaviour^38^,^39^,^40^. Such cases are not only likely for foraging animals but also for human task-switching; we continue to engage in a behaviour while it appears profitable, but disengage as the rate of return diminishes, for example, in equity markets^41^. In such situations a comparison of the current shorter-term rate of reward with the longer-term average reward rate of the environment is highly informative. The opposing, time-linked reward representations in dACC (Figs 4 and 5) allow switching behaviour as a function of the changing reward rates.

We found that activity in several brain areas including dACC, FO and striatum reflects multiple time-linked reward representations. The signs of the brain signals reflected the opposing weights of rewards in recent and distant past on choice (Fig. 4b,e) but only in dACC were these weights predictive of the weights each subject placed on recent and past rewards in their behaviour (Fig. 4c,d,f). A role for dACC in deciding based on extrapolated reward trends or expected future rewards is consistent with a recent proposal that ACC is involved in evaluating future strategies before they are executed^42^. Furthermore, neurons in macaque dACC and interconnected areas represent memories of reward rates with different time constants^18^,^19^,^22^,^43^. The behavioural role of such activity patterns has not been clear. Different reward memories could be used to adapt the learning rate of an agent to the volatility of an environment^44^. However, with an appropriate network, architecture reward tracked with different time constants could be used to extrapolate reward trends.

Standard RL models do not capture reward trend-guided choice. However, they can easily be modified to do so if the RL mechanism is able to compute an expectation of PEs, as PEs are a simple measure of reward rate change. In the same way as past outcomes influence subjects' stay rates in our experiment, expected PEs will be highest when first experiencing small rewards and only more recent rewards are high, that is, when reward trends are increasing (Fig. 3e). This is similar to successive contrast effects^14^ in which past rewards also have a negative effect on likelihood of continuous engagement in a choice. This highlights that estimating PEs is one way to track reward environments on various timescales and that reward trend-guided choice can be sufficient to successfully manoeuvre through certain types of reward environments. However, the relative importance of expected PEs (particularly in comparison with a standard RL value estimate, see also Supplementary Figs 3 and 4 for a more detailed discussion) should diminish the less useful reward trends are in guiding choice, for example, when environmental changes are abrupt and unpredictable.

Although there might be other models that capture reward trend-guided choice^45^, using the expected PE to solve this problem is appealing for several reasons. It is parsimonious because it requires only simple modifications of a standard RL model. It is consistent with recent suggestions that PEs might not solely have a role in value-updating. PEs have recently been linked to subjective well being^46^ and mood instability^47^, and it has been suggested that the PE may play a role in choice itself^25^. Furthermore, the brain regions that carried expected PE signals in our study, in particular dACC, have been linked to lower- and higher-order PEs before. For instance, activities of neurons identified recently by Bernacchia *et al*.^18^ (for example, their Fig. 3d,h,l) are influenced by past reward outcomes in a very similar manner as the expected PEs are derived from past reward outcomes in our model (Fig. 3e). These neurons, which have been found in several brain regions including the anterior cingulate cortex, are characterized by a double exponential function in which recent rewards have a sign-reversed influence on activity compared with rewards in the distant past, mirroring the behavioural and neural effects of past rewards we have found. Finally, expected PEs are also consistent with recent suggestions that dACC plays a role in predicting the PEs of lower computational layers^48^.

In agreement with the first neural analyses (Fig. 4) expected PEs were found in dACC (Fig. 5). The ventral striatum, by contrast, signalled standard PEs^21^,^24^,^49^. Standard PE signals may feed in a cortical network, arguably comprising dACC that computes a longer-term estimate of PEs. Moreover, we found a graded transition from representation of expected PEs to choice moving from posterior to more anterior dACC and into vmPFC (Fig. 5c), reminiscent of similar functional gradients in the prefrontal cortex^50^,^51^. The finding that dACC activity was related to both reinforcement history-derived variables and ensuing choices further supports the contention this area represents and translates reward history representation into a choice-related representation, as had also been suggested by the correlation between dACC neural and behavioural beta weights (Fig. 4c,d,f). In both analyses as well as in previous reports^28^,^29^,^30^, choice-related representations were present in dACC beyond difficulty signals. Moreover, choice-related signals in vmPFC and dACC were aligned in this experiment; both areas are more active for stay compared with leave choices and dACC activity increases with evidence for staying. In previous experiments, vmPFC activity was positively related to the value of staying versus discarding a current choice, while dACC was positively related to exploring versus exploiting a choice^28^,^34^,^52^,^53^,^54^. However, while the value of the stay-option in the previous experiments was relatively well known and stable, it had to be inferred in the current experiment because the future value of a patch is, by design, different from its past value. The leave-option was, on the other hand, pre-learned and stable. In other words, the outcome uncertainty for the stay-option is, unusually, higher than that for the leave-option. One hypothesis that integrates the present results with previous findings is that dACC codes value in a framework tied to pursuing the more uncertain option; by contrast, vmPFC may code the value of staying with the current or default choice^28^,^34^,^55^,^56^. The two reference frames are aligned in the current experiment but have been opposed in previous experiments.

## Methods

### Subjects

Twenty-two subjects participated. One left the experiment as a result of claustrophobia. Another was excluded from data analysis due to excessive motion (final sample: 20 subjects; eight female; aged 21–32). All provided informed consent. The study was approved by the Ethics Committee of Oxford University (MSD-IDREC-C1-2013-095). Subjects received £20 as a show-up fee and a fraction of £15 depending on task performance.

### Experimental design

In each of 90 trials, subjects proceeded through a patch consisting of reward and non-reward events (800 ms each). Between events, a fixation cross was presented and button presses led to the next event. After time step 15, 16 or 17, the subjects were offered the choice to stay for longer in the patch or to leave to a default environment with a known, stable reward rate (leave-stay decision; LSD). This meant that participants encountered LSDs at approximately similar positions in time on each occasion that they explored a new environment; however, the presence of some variability in LSD timing precluded precise anticipation of the LSD time by participants. Moreover, the analyses do not focus on activity that is simply linked to the main effect of LSD occurrence but to activity that is parametrically related to the reward experience before the LSD and that allowed the subjects to make inferences about the reward likely to be received after the LSD. The design of the trials was based on 18 monotonic reward rate curves, nine increasing and nine decreasing, from each of which five unique sequences of reward/non-reward events were derived (Fig. 1). The key manipulation was to assemble a set of patches such that the behavioural and neural effects of recent and past reward rates could be dissociated. The reward rate of a reward event was its reward magnitude divided by the number of time steps from the previous reward event or from the start of the sequence (time delay; Fig. 1c,d). The reward magnitude was indicated by the height of a golden texture (‘gold bars') within a box presented on the screen. The time delay between reward events ranged between two to six time steps. On every trial, the LSD was, without time jitter, preceded by a reward event at the last time step of the sequence to keep the recency of the last reward event with respect to the LSD constant. Note that this meant that the last time step was always rewarded and the second last time step was never rewarded. Each LSD began with a 2,000 ms ‘choice phase' indicated by a question mark on screen (referred to as ‘LSD' in time-course plots). Subjects then responded and their choices were highlighted in yellow for 800 ms (Fig. 1e). The left-right locations of ‘stay' and ‘leave' buttons were randomized. After LSDs, the subjects continued through a sequence of 15 additional time steps. For stay choices, the underlying previous reward curve continued linearly based on its slope at the time of the LSD. If subjects decided to leave to the default environment, rewards were delivered at a fixed reference reward rate (that is, the ratio of reward magnitude and time delay was identical for all reward events of the sequence) that subjects knew well from prior practice sessions. Although the specific event sequence differed between runs of the default environment, the sum of the rewards encountered there (leave value) was constant. For LSDs, subjects had to decide whether the future value of the patch, the sum of reward encountered when further committing to it (stay value), was higher or lower than the stable leave value. On 66% of increasing patches, the stay value was higher than the leave value, while on 66% of decreasing patches, the leave value was higher than the stay value. Subjects received ‘bonus points' for making the better choice in proportion to the absolute difference between the stay and leave values and this determined the performance-dependent monetary payoff subjects received at the end of the fMRI session. Optimal choices were defined as the ones with the higher payoff. Subjects received feedback about their accumulated bonus points after 1/4, 2/4 and 3/4 of the experiment. Finally, 60% of the trials were truncated after the LSD to shorten the experiment and to minimize choice feedback.

### Reward sequences

The generation of actual reward sequences from the theoretical reward rate curves was an iterative process. Beginning at the last time point of the curve (because the last event before a LSD or the end of a patch was always a reward event), a reward magnitude and a reward delay were randomly chosen under the condition that they conformed to the reward rate indicated by the reward rate curve at that time point. The chosen reward delay determined the time point of the reward event preceding this event. For this, again, reward magnitude and reward delay was randomly chosen under the condition that it they conformed to the reward rate indicated by the reward rate curve at that time point. The threshold for ‘conforming to the reward rate curve' was initially set to 5% of the reward rate given by the curve; however, it was expanded in 0.00001% steps in case no solution could be found. Note that for our analyses, it did not matter how closely the reward rates of the reward events in the sequence satisfied the reward rates originally intended for the curves because all parameters used in our analysis were derived from the actual reward sequences and not from the reward rate curves. In other words, the reward rate curves that we show and use to guide all analyses of behaviour and neural activity are the ones that were established empirically at the end of this iterative process.

### Training session

Subjects experienced a 60–70 min training session on the day before scanning and a 15–20 min training period directly before the scan. Both training sessions comprised familiarization with the default environment and a version of the experimental task with choice feedback (number of bonus points earned or missed) after each trial (Supplementary Fig. 1).

### Reinforcement-learning models

To compare subjects' choices with a learning algorithm that integrates reward rates with a single time constant we devised a standard Rescorla-Wagner reinforcement learning model (RL-simple)^23^. More complex models are based on RL-simple (see below).

At the beginning of a patch, a value estimate was set to the average reward rate experienced in the task up to that point and was zero for the first trial. The value estimate was then updated by the outcome of each time step of the patch sequence. Therefore, the number of value updates in a patch before LSD was equivalent to the number of time steps in the sequence. The value estimate was updated using a learning rate *α* fitted for every subject:


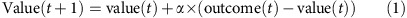


The size of the outcome was zero or positive, and reflected the reward magnitude encountered at a time step. The value estimate at the time of the LSD (*t*=LSD), that is, after the last event of the sequence, was used as the decision variable (DV):


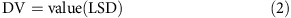


The DV, representing the value of staying in a patch, was compared with the value of the default patch to determine the model's choice. The probability of staying in a patch was calculated with a softmax equation:


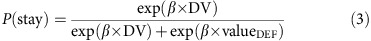


*β* is the inverse temperature of the softmax function and the constant value_DEF_ represents the value of the default environment. Note that modelling value_DEF_ as a free parameter means we do not use a RL mechanism to learn the value of the reference patch. Given that the reference patch is pre-learned and only very rarely encountered in the actual experiment (60% of trials end directly after the LSD), we determine the value each subject assigns to the reference patch empirically by treating value_DEF_ as a free parameter that is stable over the course of the experiment. Value_DEF_ was used in all RL models. We derived the choice probability from the stay probability on each trial:


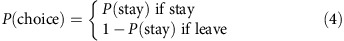


Overall, the free parameter set *θ* comprised *α*, *β* and value_DEF_. We fitted these parameters for every subject by minimizing the negative log likelihood (nLL) over all trials *N*, given a set of parameter values:


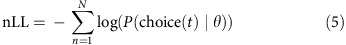


In further analyses, we expanded RL-simple to account for the reward rate trend-guided choices observed. For all expanded models (except illustrative models, see below), we fitted free parameters for each subject individually. The Softmax function used including the use of value_DEF_ (equation (3)) and fitting (equations (4 and 5), except the precise parameter set *θ*) were identical to RL-simple.

For the simplest extended model, RL-simple+lastPE, all features were the same as in RL-simple, except the DV. The DV included in addition to the last value estimate the PE of the last time step weighted by an additional free parameter (PEweight):


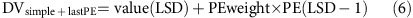


In sum, the free parameters for RL-simple+lastPE comprise *α*, PEweight, *β* and Value_DEF_.

Lastly, we constructed an average reward rate learner (RL-avgRR) that consists of two hierarchically organized RL mechanisms. First, we used a standard RL mechanism identical to the RL-simple model described above (RL-avgRR_part1_). The value estimate (value) of RL-avgRR_part1_ is updated at every time step using a learning rate *α* and a PE:


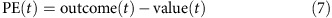



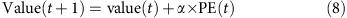


A second component of the new model, a parallel RL mechanism (RL-avgRR_part2_), learns outcomes attenuated by value using an PE_expected_ (contrasting effectively recent and longer-term past rewards), which can also be seen as learning PEs:


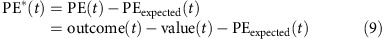



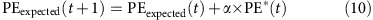


PE_expected_ at the time of choice (that is, after the last time step) was used in the DV:


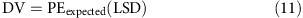


Note that for the last PE* of RL-avgRR_part2_ before choice, we fitted a separate learning rate as an additional free parameter (*α*-lastPE) allowing the model to calibrate the relative weight of past relative to the most recent reward history. This makes the model similar to the separate effect of the last PE in RL-simple+lastPE. This was performed to account for particularities in our outcome sequences (a reward outcome was never received at the second to last time step, while a reward outcome was always received at the last time step) and to make the RL-avgRR model comparable to RL-simple+lastPE. Similar to the other models, at the beginning of each trial, both value and PE_expected_ were set to the average reward rate experienced in the task up to that point. We used the same free parameter *α* for the learning rate of the basic and higher-order RL mechanism in the model (see Supplementary Note 1 and Supplementary Fig. 3 for supplementary models). Therefore, the free parameters for RL-avgRR comprise *α*, *α*-lastPE, *β* and value_DEF_.

Finally, we use an illustration to show the temporal integration function of RL-avgRR and compare it with RL-simple. We derived the functions that define the weights of past PEs and outcomes in PE_expected_ at the time of choice, (that is, the DV of RL-avgRR). For a standard RL model (for instance, RL-simple), the value estimate can be mathematically decomposed into a sequence of *n* weighted past outcomes:


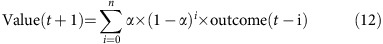


Considering that RL-avgRR learns based on simple PEs applying this formula results in (‘PE' and ‘value' refer to components of RL-avgRR_part1_ in the following equations (13, 14, 15, 16, 17)):


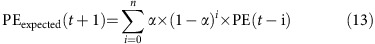


For our illustration (Fig. 3f), we show this function over a sequence of 15 time steps (*n*=15) using the median learning rate of avgRR (*α*=0.183, Supplementary Table 1). For simplicity, we ignored the fact that the learning rate of RL-avgRR_part2_ at the last time steps is decreased (median *α*-lastPE*=*0.134). To define the contribution of the outcomes rather than the PEs, the formula can be further broken down into:


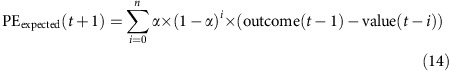


By using equation (12) with equation (14) this results in


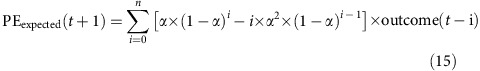


In Fig. 3e,f we illustrate equation (15) and equation (13), respectively, using the median learning rate of RL-avgRR. In Fig. 3c we illustrate equation (12) for the median RL-simple learning rate of 0.471. In Fig. 3d the weights equal the learning rate of RL-simple (but normalized, see below).

As a supplement to the functions in Fig. 3c–f describing the temporal integration dynamics of RL-avgRR and RL-simple, we ran two RL models, fitted on the whole group of subjects for illustration (grey bars in Fig. 3c–f). Both models are identical to RL-simple and only deviate with respect to the calculation of the decision variable. The models explain choices reduced to the weighted history of PEs and outcomes, respectively. For the PE illustration model (Fig. 3d,f), the DV was calculated purely on weighted PEs where the same free parameter weight was assigned to sets of three PEs (the last three PEs before LSD are assigned the same weight, and so on) using five free parameters *w*_1–5_.


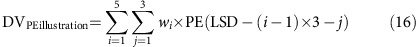


Grey bars in Fig. 3d,f show the fitted parameters *w*_1–5_. The illustration of the outcome history weights (grey bars in Fig. 3c,e) was run in the same way using the DV:


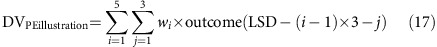


Grey bars in Fig. 3c,e show the fitted parameters *w*_1–5_. For the two illustrative models as well as the PE and the outcome functions, all parameter weights were normalized by their absolute sum for display purposes.

### Behavioural and RL model analysis

We used two complementary parameterizations of reward history. Our first behavioural GLM (Fig. 2a) comprises the reward rates in discrete time bins moving back in time relative to the LSD. For each of five reward bins, the rewards received on three consecutive time steps were averaged. Therefore, overall, the reward bins covered 15 time steps, which was the minimum length of a patch. This resulted in five parametric regressors that represented reward rates for each of five time bins before LSD:

*Behavioural GLM1*: LSD-13-15, LSD-10-12, LSD-7-9, LSD-4-6 and LSD-1-3 (Figs 2a and 3g), where LSD-1-3 represents the reward rate of the bin corresponding to the last three time steps before LSD and LSD-4-6 represents the reward rate of time steps 4–6 before LSD, and so on. For this and all other behavioural GLMs, regressors were normalized (mean of zero, standard deviation of 1).

Our second set of parameterizations of reward history separates past rewards in lastRR and avgRR as well as lastRR−avgRR. LastRR is the reward rate of the last reward event, that is, the magnitude of the last reward event divided by the number of time steps to the second last reward event. AvgRR is the sum of reward magnitudes of all reward events in a patch (including also the last reward event) divided by the number of time steps until the LSD. Hence, avgRR includes lastRR. We used the following GLMs:

*Behavioural GLM2*: lastRR and avgRR (Figs 2b and 3h).

*Behavioural GLM3*: lastRR−avgRR (Figs 2c and 3i and Supplementary Fig. 2d).

*Behavioural GLM4*: lastRR, lastRR−avgRR (Supplementary Fig. 2c).

All four behavioural GLMs were applied to subjects' actual choices and RL-predicted choices. Essentially, we compared the stay probabilities from the RL model (*p*(Stay) from equation (3)) with subjects' actual stay rates. We did this for RL-simple (Fig. 2) and RL-avgRR (Fig. 3g–j). To use the GLMs on RL-predicted choices, for every subject, we simulated binary choice behaviour from the RL stay probabilities via a binomial distribution and then applied a GLM to the simulated choices. We repeated this binary choice simulation and application of GLM 1,000 times per GLM and subject and averaged the resulting beta weights per subject to determine the influence of the regressors on RL-predicted choices. Error bars on the RL's beta weights always indicate s.e. between subjects. For Figs 2d and 3j, we binned trials by the categorical type of reward rate trend (increasing or decreasing) and the optimal choice (stay or leave), calculated the actual stay frequencies and compared them with subjects' RL-predicted stay probabilities (*p*(Stay) from equation (3)) averaged per bin. Note that in 66% of increasing trials it was optimal to stay, while in 66% of decreasing trials it was optimal to leave (optimal in the sense of maximal payoff).

Moreover, we constructed a measure of response selection or task difficulty using behavioural GLM4. For every subject, we combined individual beta weights of lastRR and lastRR−avgRR with the parameter values of lastRR and lastRR−avgRR in a logistic GLM to predict the stay probabilities on every trial. We also included the subject-specific constant. For all subjects, we derived a measure of task difficulty for each trial:


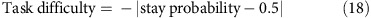


Behavioural data were analysed with SPSS and MATLAB, using paired *t*-tests and repeated measures analyses of variance, including Greenhouse–Geisser correction where appropriate.

Details on MRI data acquisition and analysis are presented in the Supplementary Methods.

### Data availability

The data that support the findings of this study are available from the corresponding author upon request.

## Additional information

**How to cite this article:** Wittmann, M. K. *et al*. Predictive decision making driven by multiple time-linked reward representations in the anterior cingulate cortex. *Nat. Commun.* 7:12327 doi: 10.1038/ncomms12327 (2016).

## Supplementary Material

Supplementary Information Supplementary Figures 1-7, Supplementary Tables 1-2, Supplementary Note 1, Supplementary Methods and Supplementary References
