# Supplementary material for: Predictive decision making driven by multiple time-linked reward representations in the anterior cingulate cortex
Source: Nat Commun. 2016 Aug 1;7:12327. doi: 10.1038/ncomms12327 (PMC4974652; doi:10.1038/ncomms12327)
Supplement: Supplementary Information — Supplementary Figures 1-7, Supplementary Tables 1-2, Supplementary Note 1, Supplementary Methods and Supplementary References [file ncomms12327-s1.pdf]

Supplementary Figures:

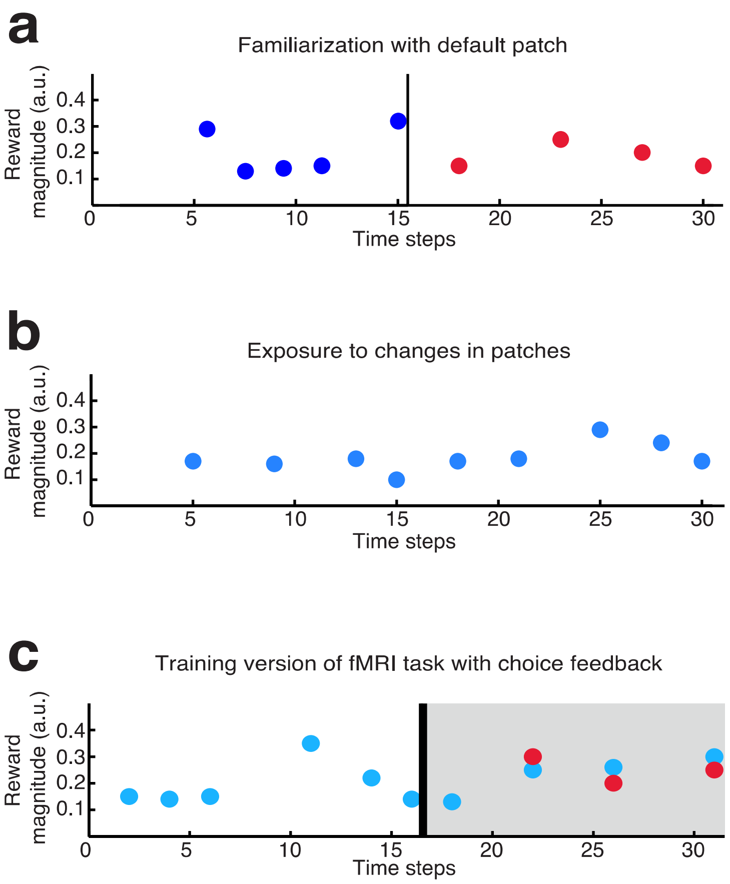

Supplementary Figure 1. Supplementary task description relating to Figure 1. Illustration of the training schedule prior to fMRI scanning. Subjects were trained on the day before fMRI scanning and on the day of scanning, immediately preceding the experiment. The full training session (only performed on the preceding day) comprised three parts. (**a**) Familiarization with default patch (10 trials). In each trial, alternatingly, subjects experienced a stable default patch (red) and length-matched experimental patch (blue). Dots represent reward events of a given reward magnitude and the vertical line represents the cued transition to the other patch. After both patches (i.e. after time step 30), subjects were asked to indicate which environment had contained more rewards (i.e. had the overall higher reward rate). (**b**) In part two, subjects experienced three patches. They were told that a patch's payoff changed monotonically and that this becomes apparent when paying attention to both the reward magnitudes and reward delays of reward events. Subjects pressed through each patch three times with varying instructions. The instructions were to pay attention to 1) the change in reward magnitudes (ignoring delays) 2) the change of reward delays (ignoring reward magnitudes) and 3) the change in both magnitudes and delay. This part contained no decision. The reward sequence displayed in (b) was taken from an increasing reward rate curve (note the increasing payoff of the reward magnitude to reward delay ratio: the reward magnitudes increase and/or the delays between rewards decrease). (**c**) In the third part, subjects were asked to do a training version of the experimental task including leave-stay decisions (18 trials). Notably, they had the chance to experience the experimental patch (blue) or the default patch (red – this was only presented after the LSD when participants opted for the default option) and they were given performance feedback after each trial. The vertical line represents the time of the LSD and dots in the grey area represent reward events from the experimental patch (blue) and the default patch (red). Subjects experienced only one or the other on any given trial. For performance feedback, subjects were told how many bonus points they had earned or missed depending on their choice (see Experimental Procedures). Bonus points were proportional to the overall payoff of the chosen compared to the unchosen patch after LSD. Hence, a positive number indicated a correct choice (earned points) and a negative number an incorrect choice (points missed). This step allowed subjects to discern which aspects of the reward environment were predictive of correct choices. The reward sequence displayed was taken from a slightly decreasing reward curve (note the decreasing payoff of the reward magnitude to reward delay ratio: the reward magnitudes decrease and/or the delays between rewards increase). (**a,b,c**) In sum, the training session gave subjects the opportunity to memorize the

stable default patch, to grasp the structure of the reward environments and to learn how to make correct decisions. On the day of scanning, a shorter training session took place to refresh the experimental instructions. In the fMRI scanner, subjects had no opportunity to learn from direct choice feedback and 66% of trials were truncated after the leave-stay decision, discouraging changes in choice strategy during the fMRI session.

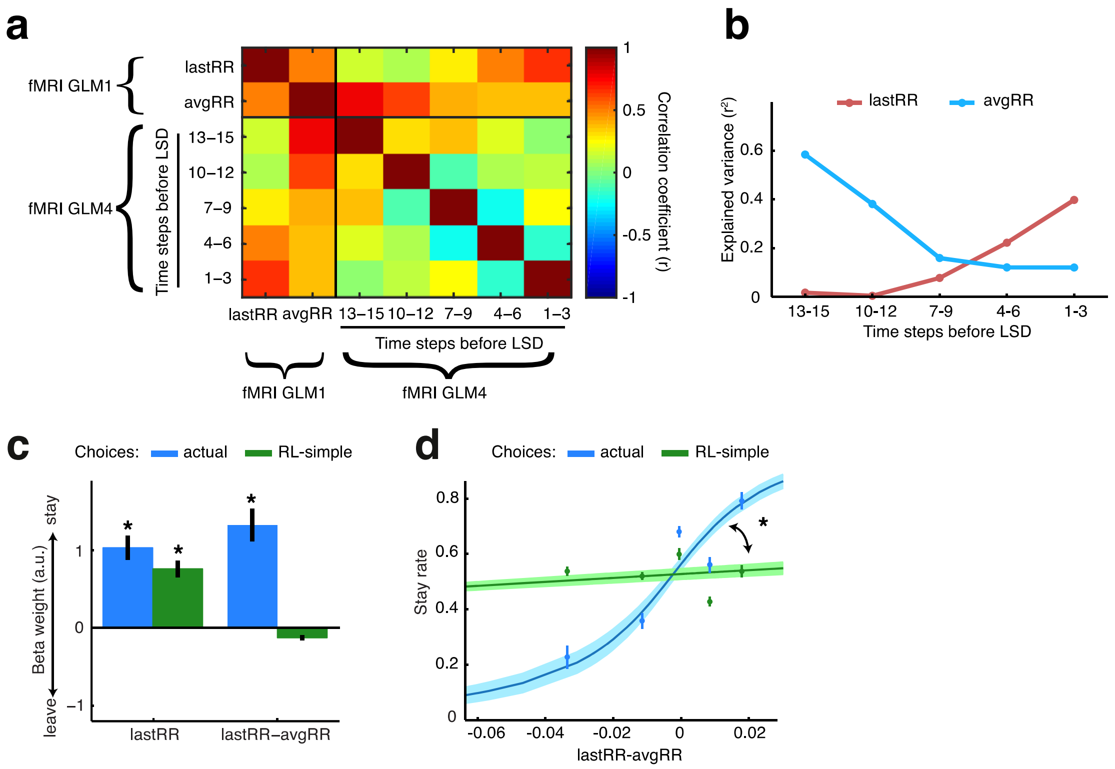

Supplementary Figure 2. Supplementary behavioral results relating to Figure 2. (**a**) Correlation matrix of parameterizations of reward history used in two GLMS (fMRI GLM1 and fMRI GLM4). The regressors used in our two GLMs summarize reward history in complementary ways. Note that the parametric regressors within each GLM (lastRR and avgRR in fMRI GLM1 and the five time bins in fMRI GLM4) share less than 25% of their variance. (**b**) The two GLM analysis approaches are, however, complementary and so there are relationships between them. An illustration of the relation between the time bin GLM (GLM4) and the lastRR/avgRR GLM (GLM1) is provided. Each data point represents shared variance (r^2) between a reward rate in the time bin-based GLM and lastRR and avgRR, respectively (squared correlation coefficients from panel a, lower left part). Note that the underlying correlation coefficients are positive in all cases (see panel a). LastRR shares more variance with more recent time bins compared to earlier ones while, by contrast, avgRR is increasingly correlated with earlier time points. However, the change is gradual, reflecting the continuous structure of the reward rate curves. This illustrates that the positive effect of lastRR on choice predicts increasingly positive beta weights for recent time bins, while the negative beta weight of avgRR (Figure 2b) predicts decreasingly negative beta weights for early time bins. Graphically speaking, the continuously increasing beta weights of recent time bins in Figure 2a can be interpreted as a superposition of a positively weighted lastRR correlation profile and a negatively weighed avgRR correlation profile. Compare also with Figure 1b that shows that reward rate curves differ more early in a patch as a function of their trajectory compared to later. This asymmetry makes it easier to dissociate lastRR from avgRR in behavioral and neural analyses, because it reduces their correlation. (**c**) Complementary GLM to Figure 2b. We used a GLM with lastRR and lastRR-avgRR as regressors. Subjects (blue) were influenced by both lastRR (t_{19}=6.64; p=2*10^{-6}) and its relation to earlier reward rates (lastRR-avgRR: t_{19}=6.23; p=5*10^{-6}). RL-simple (green) predicted correctly that choices would be guided by lastRR (t_{19}=7.03; p=1.1*10^{-6}). However, it failed to reflect that subjects tended to stay when reward environments had become better over time (negative effect of lastRR-avgRR; t_{19}=-3.65; p=0.0017). (**d**) Supplementary description of Figure 2c. We sorted all patches by their lastRR-avgRR and then fitted a softmax function to assess whether subjects' stay rates were positively influenced by the reward rate trend. Comparing the inverse temperature of the softmax functions between subjects' actual stay rate (blue) and RL-predicted stay rate (green), we found that subjects modulated their choice behavior significantly more according to lastRR-avgRR than a simple RL model predicts (t_{19}=7.3; p=6*10^{-7}). Overlaid are binned choice probabilities that were calculated in five continuous bins of 18 trials, sorted by lastRR-avgRR. For actual choices, the choice probabilities reflect choice frequency, for RL-predicted choices, the choice probabilities reflect p(stay) from equation 3 of the Methods section describing the RL model. (**, p<10^{-5}; *, p<0.005, one-sample t-tests (panel c) and paired t-tests (panel d) ; error bars and shaded error bars are s. e. m. between subjects).

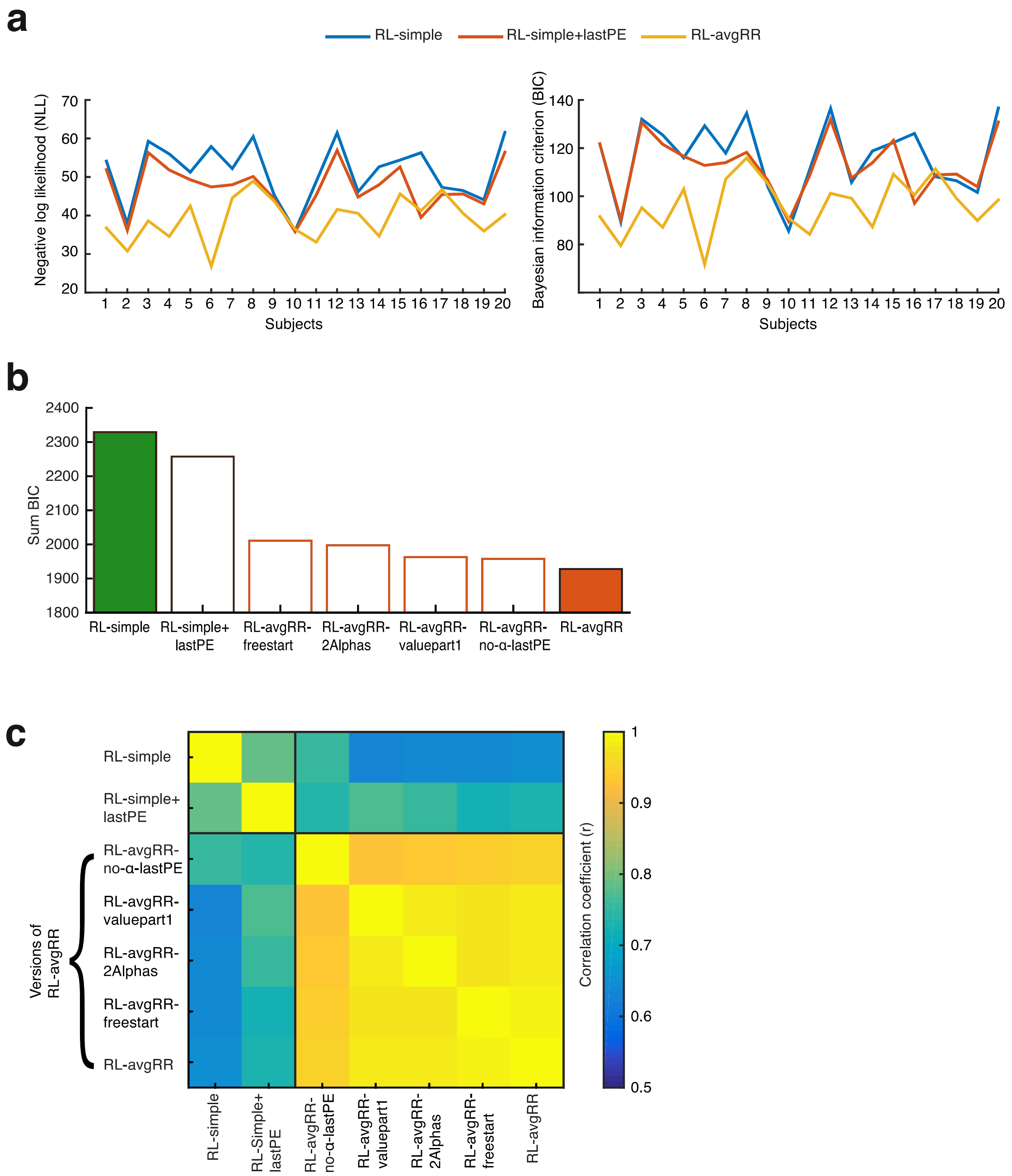

Supplementary Figure 3. Supplementary RL results relating to Figure 3. (**a**) Negative log likelihoods (left) and Bayesian information criterion (BIC) scores (right) of fitted models shown for all subjects. Smaller values indicate better model fit. (**b**) Supplementary model comparison including the three main models from Figure 3 (RL-simple, RL-simple+lastPE and RL-avgRR) and four modified versions of RL-avgRR (see Supplementary Note 1 for a description). The group sums of the Bayesian information criterion (BIC) are shown for each model sorted by BIC value. Smaller values indicate better model fit. In the modified RL-avgRR models (white bars with orange borders), we changed a single aspect of RL-avgRR to evaluate how this change affected the model dynamics. We found that all RL-avgRR models explained behavior in a robustly better manner than the two reference models (RL-simple and RL-simple+lastPE). This demonstrates that neither of the modified aspects in isolation is crucial for RL-avgRR to work in our experiment and suggests that the RL-avgRR's goodness of fit is indeed related to the model's core feature of calculating expected prediction errors (see main text). (**c**) Correlations of decision variables across models shown in panel b. Correlations are averaged over subjects using the unsigned correlation coefficients. As expected from the model comparison, the choice computations performed by all versions of the RL-avgRR model are highly similar (all correlations above 0.929) but distinct from the two reference models.

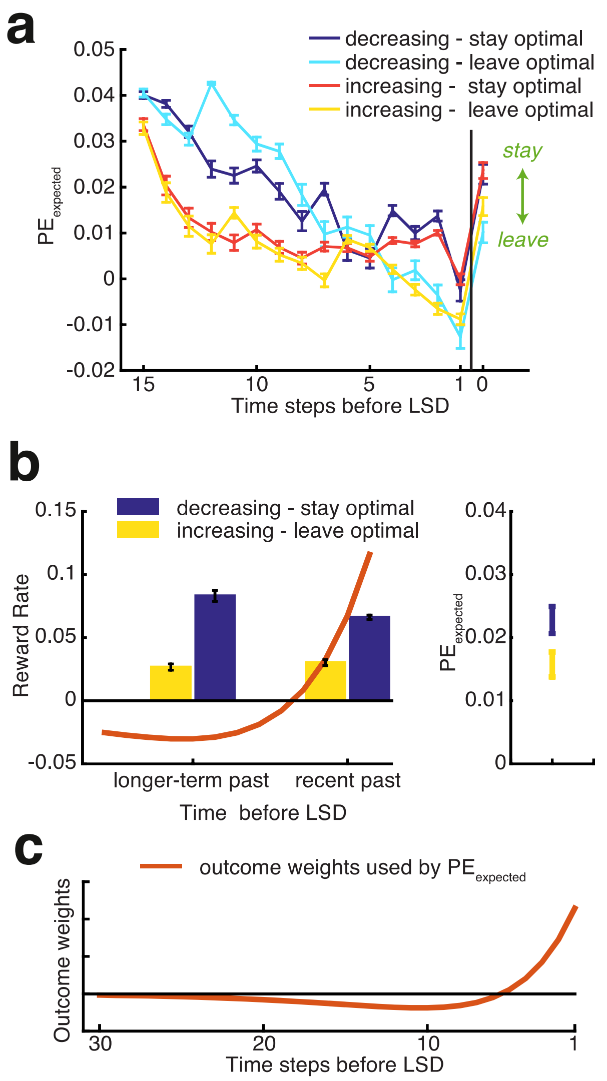

Supplementary Figure 4. Supplementary RL results relating to Figure 3. This figure explains a particular feature of RL-avgRR: the fact that a simple value estimate is not included in its decision variable. The decision variable instead consists only of $PE_{expected}$ at the time of choice. While we show that a memory of previous PEs can be used to decide according to reward trends of the environment, PEs are also influenced by the absolute reward levels in a trial. Hence, it is not necessary to use a simple value estimate in the decision variable in addition. This can be explained in terms of the learning process in a trial, where information about absolute reward levels is initially reflected by the reward trend estimate (a) or, in a complementary way, in reference to the precise pattern of the outcome weights used by RL-avgRR (b). A critical feature for both explanations is the time horizon over which rewards are experienced. Due to the relatively small learning rates (median of 0.183) there is no need to add a simple value estimate to the model's decision variable. (c). (**a**) This figure shows the development of $PE_{expected}$ over time separated by both reward trend (increasing/decreasing) and optimal choice at decision point (stay/leave; the optimal choice in a trial is defined as the one that leads objectively to more rewards on the current trial). Note that rewards are presented intermittently in our experiment. This explains sudden increases or decreases in the temporal evolution of $PE_{expected}$, particularly during the last two time steps (a reward is always presented on the last time step and is never presented on the second to last time step, see Methods. We did this to ensure that recent information about reward rates was available at the decision point in a similar manner in both increasing and decreasing environments in which it was optimal to stay in which it was optimal to leave. If this had not been then done then whether reward was delivered at the last time step or not could have contaminated our model estimate). For this, the important feature of this plot is the general development of the decision variable (i.e. $PE_{expected}$), rather than interpreting each time step separately. The figure shows how early in a trial (left hand side), $PE_{expected}$ is high or low as a function of the type

of reward environment (increasing or decreasing), but later on as a function of the optimal choice (stay or leave; right hand side). In detail, early in a trial, $PE_{expected}$ reflects learning about the average reward levels of the environments, which are initially higher in decreasing patches. In other words, $PE_{expected}$ is high at first in decreasing patches as these are initially experienced as increasing as the agent first gathers experience of the environments. Vice versa, the increasing patches are experienced as decreasing relative to subjects' initial expectations. Expressed in terms of simple prediction errors, this means that early in a trial positive simple PEs predominate in the decreasing environments; the model learns that the current rewards are better than what is expected on average. This is how the model's $PE_{expected}$ initially captures the absolute reward levels of the environment. From that point on, additional rewards modify $PE_{expected}$ in the direction of the reward rate trend (approximately the last eight time steps). Therefore, $PE_{expected}$ decreases more in patches that are more strongly decreasing (and leave is the optimal choice) compared to patches that decrease but not to such a strong degree (stay still optimal). In contrast, $PE_{expected}$ increases more strongly for patches with a stronger increase in reward rates (and stay is optimal) compared to patches were reward rates increase, but not sufficiently enough (and leave is optimal). In summary, RL-avgRR scales initially with absolute reward levels in a patch in terms of their positive or negative deviation from a longer time average reward rate (i.e. their initial trend). This estimate is then modified by the later increase or decrease of the reward rates in the patch, i.e. reward trends at decision point (increasing/decreasing). Therefore, the model's $PE_{expected}$ is related to both the reward rate trend as well as the absolute reward levels in our experiment. Moreover, there are a few things to note in this figure. First, subjects are naive with respect to the environment when they enter it. If this had not been the case and subjects did not have to learn initially about the reward levels in increasing and decreasing patches, then the early PEs could not be used to approximate absolute reward levels. However, as long as the starting value of the model reflects a relatively constant feature of the past reward history (i.e. is not initialized as the first reward rate of the specific patch), relative preferences based on developing trends and higher and lower absolute reward levels can be reconstructed in terms of the relative deviation from that initial constant. This is also why different starting values lead to similar model fit (see Supplementary Fig.3b). Second, note that as learning about the environments proceeds, all prediction error estimates ($PE_{expected}$) decrease. This effect is superimposed on the other effects described before (initial clustering of $PE_{expected}$ by increasing/decreasing, then by stay/leave). Third, the fact that rewards are presented intermittently in our experiment (as well as in most probabilistic learning tasks) leads to relatively low learning rates for RL-avgRR (median 0.183), reflecting integration over many time steps to get a stable value estimate. (**b**) A complementary explanation of why PEs also contain information about what we called absolute reward levels is related to the precise pattern of outcome weights used by RL-avgRR in our experiment (orange line in left panel; from $PE_{expected}$ as in Fig.3e). We illustrate this for trials of our experiment in which decisions have to be made against the reward rate trend, as those could not be made correctly without information about absolute reward levels. We show the average reward rates of reward events binned by recency (median split in recent past and longer-term past relative to choice) for trials in which it was optimal to leave although they were increasing (yellow) and in which it was optimal to stay although they were decreasing (dark blue) (left panel). Optimality was defined as the choice that led to objectively more rewards on the current trial, as in panel (a). For these two trial types, decisions had to be made based on experienced reward levels and against the reward trends. That the model could do this is clearly reflected by the higher decision variable of RL-avgRR (i.e. $PE_{expected}$) for decreasing/stay compared to increasing/leave trials (right panel; see also Fig.2d). A complementary explanation to above is to illustrate the outcome weights directly. For this we need to look more closely at the model's positive (orange line, right hand side) and negative weights (orange line, left side) of recent and past outcomes, respectively. These weights are not balanced (i.e. they do not sum up to zero) but instead positive weights are stronger than negative weights within the time frame of approximately 16 time steps of each trial. Hence, there is a stronger positive than negative weighting (the relation is approximately 4:2.5 for positive compared to negative weights for the function shown in this figure). This distribution of weights is caused by an interaction between the time horizon studied and a relatively low learning rate. The stronger positive influence of recent compared to the negative influence of longer-term past rewards enables the model to differentiate between environments with varying absolute reward levels. In other words, even though there was a negative contrast effect that higher levels of past rewards exerted on the valuation of recent rewards, this contextualization was not complete (see panel c for a case where the contextualization is complete and an additional simple value estimate is needed to guide choices). The following numeric example gives an illustration how the imbalanced weight set can be used to differentiate between high and low reward environments in the absence of a reward trend. Imagine a stable environment where one reward was delivered in the negative past time window and one reward was delivered within the positive time window (for instance

think of the yellow bars in the plot having both a reward rate of 1). Imagine also the positive and negative part of the orange line were just two values, a positive one and a negative one, summarizing the overall weight during that recent and longer-term past time period (for instance -2.5 and 4, as is actually the ratio for the orange line). $PE_{expected}$ could then be calculated as the (negative) sum of the past rewards times the negative past weight (-2.5) plus the recent rewards times the positive recent weight (4). In this case, $PE_{expected}$ of RL-avgRR would be 1*(-2.5) +1*4=1.5. In an environment with a higher stable reward rate (for instance the dark blue one), e.g. two rewards per time window, $PE_{expected}$ would be 2*(-2.5) +2*4=3. Consequently, $PE_{expected}$ and thus the decision variable is higher (3 compared to 1.5) for the environment with the higher stable reward rates (dark blue). Note that in this example the resulting estimates are positive, just as it was predominantly the case in our experiment (positive values in right panel), reflecting the imbalance of stronger positive compared to negative outcome weights. This example illustrates how $PE_{expected}$ is able to differentiate between stable environments with varying absolute reward rates. In other words, the imbalance of positive and negative weights means that the RL-avgRR decision variable contains not only information about the later reward trend but also the non-changing part of the reward environment, as this components has not been completely balanced out. In other words, given the learning rate and the number of time points over which people experienced the environment, the outcome experienced early and late in the environment were not balanced in regard to the beta weights. **(c**) For completeness, we also want to outline example cases where it is likely to be important to include an additional simple value estimate in the decision variable to capture absolute reward levels. These are likely to be ones where learning of the environment occurs over a very long time period (longer than in our experiment). To illustrate this, we show the outcome weights used by RL-avgRR's $PE_{expected}$ expanded over a longer time period (same function as shown in Fig.3e, but longer time frame). Here, the negative tail of the function (orange) on the left hand side converges over 30 time steps. In this case, all outcome weights sum up to zero. In other words, positive recent and negative past weights cancel each other out. This means that $PE_{expected}$ in a completely stable environment would be zero, any decrease in reward rate recently would mean a negative $PE_{expected}$ and any recent increases would mean a positive $PE_{expected.}$ In such a setting, $PE_{expected}$ would only reflect the change in reward rates and not absolute reward levels. Note that even in this setting RL-avgRR would still be perfectly able to perform the computation that is the emphasis of this manuscript; it would use a longer-term memory of PEs to estimate reward trends i.e. recent changes in the reward environment. Only a simple RL value estimate (which RL-avgRR computes anyways to estimate the PEs) would have to be included in the decision variable to capture differences in absolute reward levels. Therefore, in contexts with very stable environments longer than participants learning horizon or when the initial reward level is known to subjects preceding a trial, the inclusion of a simple RL's value estimate could allow a model to capture both trend and absolute reward level information. However, in our experiment $PE_{expected}$ alone, without a simple value estimate, was sufficient to dissociate between environments with rich and poor but relatively stable reward rates (see panel a,b) as such reward levels had only recently been learned themselves.

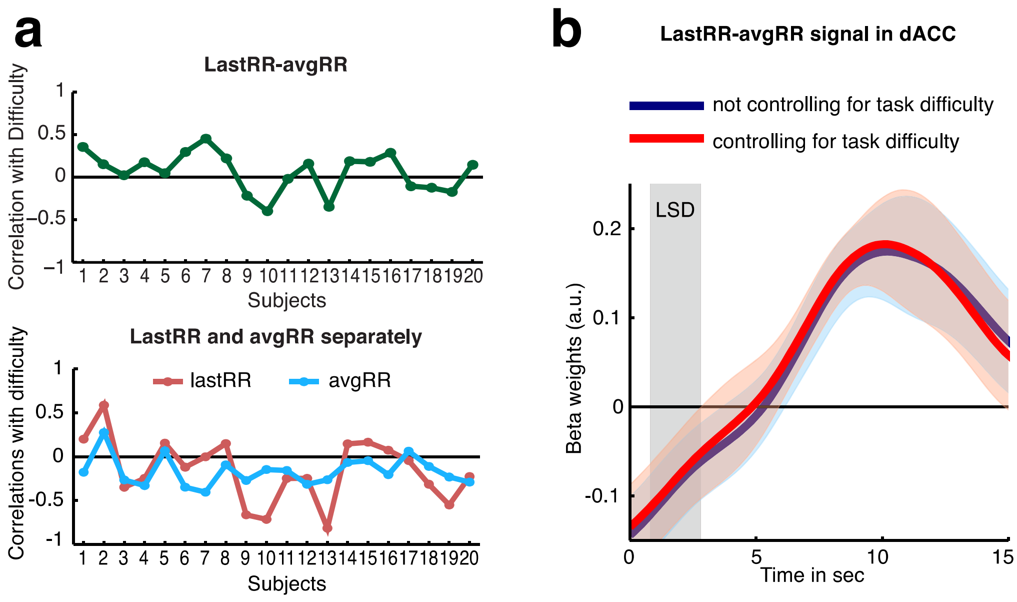

Supplementary Figure 5. Supplementary fMRI results relating to Figure 4a,b,c,d. (**a**) Neural effects of task difficulty were separable from effects of recent and past reward estimates. We calculated a trial-by trial measure of task difficulty[1] using choice predictions from one of our behavioral GLMs (Methods). Task difficulty did not correlate in a systematic way with lastRR-avgRR, the key parameter in our whole brain analyses that led us to identify dACC activity (top). The same was true for its correlations with the components lastRR and avgRR (bottom). (**b)** We calculated fMRI GLM1 (Methods) as a time course analysis time-locked to LSD and computed the effect of lastRR-avgRR in dACC (blue line). We repeated this GLM and included task difficulty as additional regressor (supplementary fMRI GLM1). Overall, the parametric modulators of the constant time-locked to the LSD in this GLM comprised lastRR, avgRR, logRT and task difficulty. The lastRR-avgRR effect after including task difficulty (red line) showed a slight, but not significant increase in peak effect size relative to the lastRR-avgRR effect from fMRI GLM1.

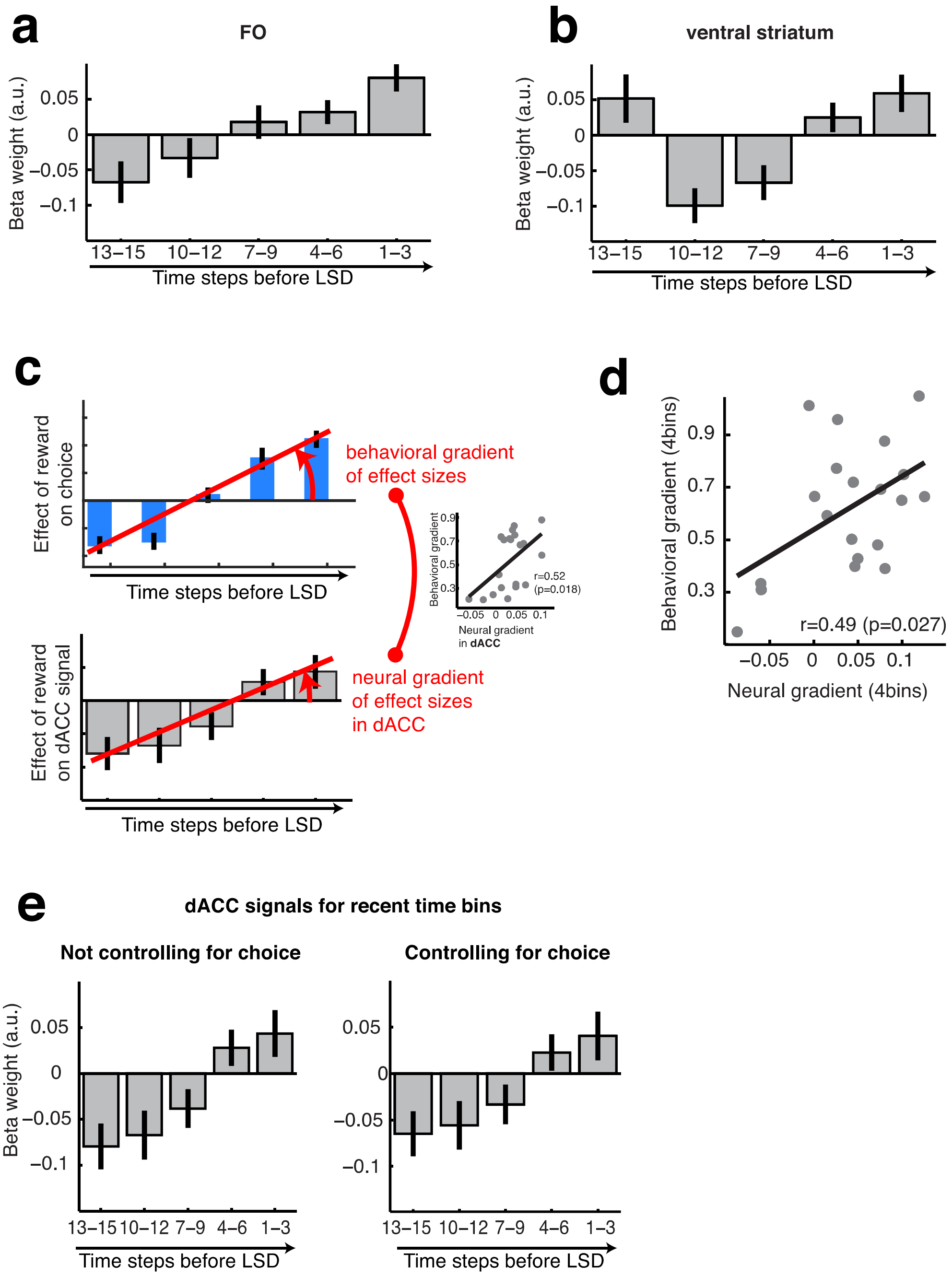

Supplementary Figure 6. Supplementary fMRI results relating to Figure 4e,f. (**a**,**b**) Result of time bin analysis of frontal operculum (FO) and ventral striatum. The analysis is analogous to the one presented in Fig.4e for dACC, where dACC showed a gradual transition from positive to negative effect sizes as reward rates became more distant in time. The same was true for FO but not the ventral striatum. To quantify similarity between the three areas, we calculated a 3 (ROI) x 5 (time bin) ANOVA and found a significant effect of time bin ($F_{2,47}$=8.39; p=3.3*10^{-4}) and an interaction effect ($F_{8,152}$=3.68; p=0.001) suggesting differing temporal gradients of neural response to reward rates (additional analysis of another ventrostriatal ROI also failed to identify monotonic changes in effects of the sort seen in ACC and FO). (**c**) Illustration of the analysis shown in Fig.4f. We used a linear regression to fit a line through each subject's behavioral beta weights shown in Fig.2a and through the neural beta weights reflecting the influence of the five time bins on neural activity. So each line was fitted on five data points. We did this separately for each ROI (dACC from Fig.4e shown here as an example). We used the slope of the fitted lines as indices of behavioral and neural gradients, respectively. Fig.4f showed that there was a correlation between behavioral gradient and dACC gradient across subjects. This was not the case for FO or the ventral striatum. (**d**) Control analysis for the correlation between dACC signal and behavior shown in Figure 4f. The correlation between temporal gradients of beta weights in dACC and in behavior remained significant, when both gradients

are calculated based on the last four instead of the last five reward rate bins (*r*=0.49; *p*=0.027; the latter analysis was done in Figure 4f). Visual inspection of the neural beta weights for all reward bins in Figure 4e and panels (a.b) of this supplementary figure may suggest that the difference between cortical areas and the ventral striatum only relates to the earliest time bin LSD-13-15. To show that the significant correlation between neural and behavioral gradient in dACC (Figure 4f) is not only dependent on this earliest time bin, we repeated the analysis shown in Figure 4f, but removed the earliest time bin from the calculation of the neural and behavioral temporal gradient. The correlation remained significant. (**e**) In another control analysis, we recalculated the time bin GLM for dACC shown in Figure 4e (shown again here on the left), but included in addition a regressor controlling for choices subjects made (supplementary fMRI GLM2). This binary choice regressor contained "1" for stay and "-1" for leave choices. The right panel shows the neural signals of the five time bins when including such a regressor. Visual inspection shows only minimal change between both GLMs. Accordingly, in a 2 (GLM) x 5 (Time bin) ANOVA, we found differences in neural response to the time bins (main effect time bin: $F_{4,76}$=4.6; *p*=0.002) but no difference between both GLMs (main effect GLM: $F_{1,19}$=2.18; *p*=0.156) and no interaction effect ($F_{4,76}$=1.12; *p*=0.318).

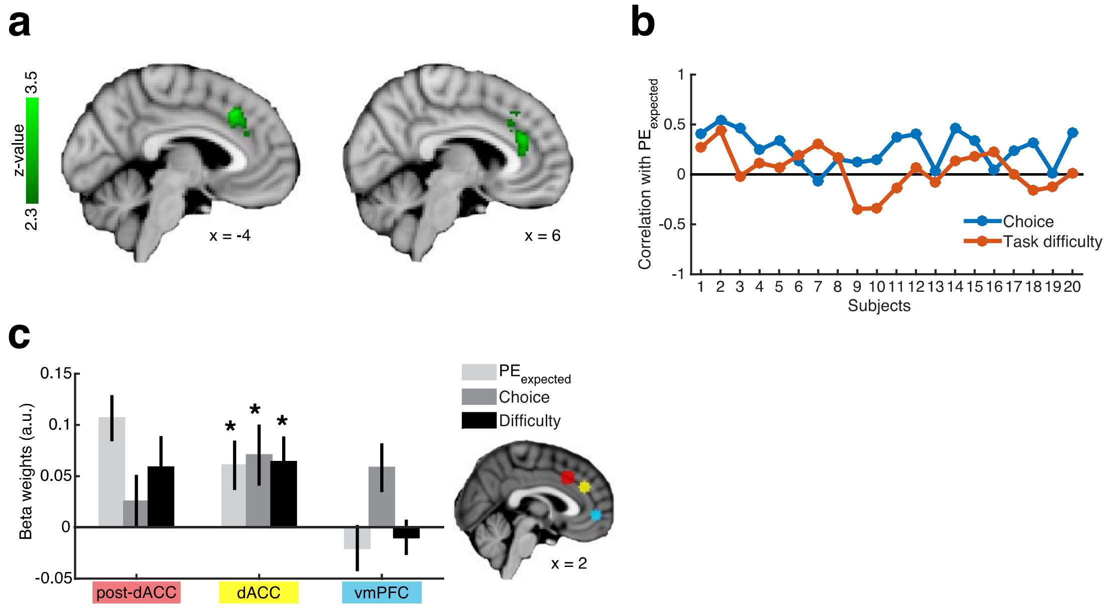

Supplementary Figure 7. Supplementary fMRI results relating to Figure 5. (**a**) Supplementary fMRI GLM3. We constructed a GLM using the same constant regressors (i.e. the regressors indicating occurrence of key trial events such as LSD time) as in fMRI 1,2 and 3. However, for this GLM, the parametric regressors time-locked to the LSD comprised only logRT and the decision variable (DV) of the RL-avgRR model, which was the value estimate of the higher-order part of RL-avgRR model at the time of the LSD ($PE_{expected}$ (LSD)). We identified a DV signal in an extended cluster in the cingulate cortex, comprising more posterior regions similar to the results in Figure 5a and also more anterior ACC regions similar to the results in Figure 4a. In addition, the DV was reflected in the BOLD response in the right dorsolateral prefrontal cortex (Supplementary Table 2). (**b**) For further analysis in Figure 5 and Figure 6, we used the expected prediction error one time point before choice ($PE_{expected}$(LSD-1), abbreviated as just " $PE_{expected}$" in the main text and in this figure. Here, we show correlations of $PE_{expected}$ with choice and task difficulty for all subjects. Choice was coded as "1" and "-1" for stay and leave trials respectively, and task difficulty was calculated as in supplementary Figure 5 (Methods). (**c**) Supplementary fMRI GLM 4, complementary to ROI analyses to Figure 5c. Using the same ROIs as in Figure 5c, we recalculated fMRI GLM 2 (shown in Fig.5), using task difficulty as an additional parametric regressor. Not unexpectedly for a brain region that computes choice variables, task difficulty had a significant effect in dACC (t_{19}=2.74; p=0.013) but not in the other two regions (postACC: t_{19}=2.02; p=0.058; vmPFC: t_{19}=-0.62; p=0.54). However, the $PE_{expected}$ and choice signals in dACC remained significant and even exhibited very small increases in effect sizes (t_{19}=2.68; p=0.015 and t_{19}=2.49; p=0.022, respectively). Note that the $PE_{expected}$ effect in post-dACC and the choice effect in vmPFC were both significant in the initial whole brain analysis that was used to first identify the ROIs but here, to avoid double-dipping, the $PE_{expected}$ effect in post-dACC and the choice effect in vmPFC, are only shown for illustration and were not further tested for significance (it is for this reason that we did not add asterisks for the pale grey bar for post-dACC and the dark grey bar for vmPFC).

Main models:

Parameter	**RL-simple**		
	α	*β*	$value_{DEF}$
Median (SE)	0.471 (0.059)	40.909 (6.239)	0.087 (0.008)

Parameter	**RL-simple+lastPE**			
	α	*PEweight*	*β*	$value_{DEF}$
Median (SE)	0.493 (0.071)	-0.308 (0.072)	57.960 (48528.45)	0.047 (0.009)

Parameter	**RL-avgRR**			
	α	*α-lastPE*	*β*	$value_{DEF}$
Median (SE)	0.183 (0.016)	0.134 (0.012)	240.46 (20.89)	0.015 (0.002)

Parameter	**RL-avgRR (fitted on all subjects)**			
	α	*α-lastPE*	*β*	$value_{DEF}$
value	0.184	0.163	161,14	0.017

Supplementary models:

Parameter	**RL-avgRR-no- *α*-lastPE**		
	α	*β*	$value_{DEF}$
Median (SE)	0.173 (0.014)	174 .643 (19.023)	0.020 (0.001)

Parameter	**RL-avgRR-2Alphas**				
	α-part1	*α-part2*	*α-lastPE*	*β*	$value_{DEF}$
Median (SE)	0.173 (0.037)	0.185 (0.048)	0.131 (0.021)	266.867 (31.398)	0.016 (0.003)

Parameter	**RL-avgRR-valuepart1**				
	α	*weight-valuepart1*	*α-lastPE*	*β*	$value_{DEF}$
Median (SE)	0.192 (0.017)	-0.030 (0.122)	0.139 (0.030)	236.565 (35.711)	0.017 (0.010)

Parameter	**RL-avgRR-freestart**				
	α	*α-lastPE*	*β*	$value_{DEF}$	*start-* $PE_{expected}$
Median (SE)	0.184 (0.016)	0.137 (0.013)	238.384 (20.987)	0.015 (0.002)	0.0233 (0.225)

Supplementary Table 1. Supplementary RL results relating to Figure 3. Summary of parameter estimates of RL models. (SE is standard error). Note in particular the smaller parameter values for $value_{DEF}$ for versions of RL-avgRR compared to versions of RL-simple. The reason for this is that while RL-simple computes standard value estimates reflecting a recency-weighted average of the

reward environment, the estimates computed by RL-avgRR reflect a contrast measure of recent and past reward rates (for a visualization of this fact, compare the weights of past rewards for these models in Fig.3c and Fig.3e). As such, they are the sum of positively and negatively weighted past rewards, resulting in numeric values closer to zero.

Contrast	Region	Peak Coordinates x/y/z (in mm MNI space)	Z-Value
last-avgRR (fMRI GLM 1)	Dorsal anterior cingulate cortex (dACC; RCZa)	6 38 28	3.93
	Right frontal operculum (FO)	34 26 -2	3.08
	Right ventral striatum	10 10 -4	3.49
Choice (fMRI GLM2)	ventromedial prefrontal cortex (vmPFC)	-2 50 -2	3.19
$PE_{expected}$(LSD-1) (fMRI GLM2)	posterior dorsal anterior cingulate cortex (post-dACC; RCZa)	2 20 38	3.82
	right inferior frontal sulcus (IFS)	38 28 22	3.82
	right inferior parietal lobule	56 -38 44	3.63
	right Thalamus	8 -10 8	3.52
$PE_{expected}$(LSD-1) (fMRI GLM3)	right inferior frontal gyrus	44 10 24	3.68
	Dorsal anterior cingulate cortex (RCZa)	-4 22 40	4.25
	right ventral striatum	8 6 0	3.65
	right frontal operculum	30 22 -4	3.66
	left frontal operculum	-32 18 -6	3.49
$PE_{expected}$(LSD) (supplementary fMRI GLM3)	Right inferior frontal junction (IFJ)	52 10 26	3.52
	anterior rostral cingulate zone (aRCZ)	-6 24 40	3.46

Supplementary Table 2. Supplementary fMRI results relating to Figure 4,5 and 6. Peak coordinates of significant clusters in fMRI contrasts ($|z|>2,3$, $p<0.05$, cluster corrected).

Supplementary Note 1 (related to Supplementary Fig.3). Supplementary reinforcement learning models were constructed based on RL-avgRR. Results relating to these models are presented in Supplementary Fig.3. Rationale and features of the modified models were the following (see also Supplementary table 1 for median parameter weights of main and supplementary models):

- RL-avgRR-no-α-lastPE: This model is identical to RL-avgRR, but does not use a separate learning rate for the last PE*. Hence, it has one less free parameter than RL-avgRR. One reason why the separate learning rate of RL-avgRR improves model fit slightly might be that the last time step before decision (the time step to which the additional learning rate relates) is always rewarded in our experiment. This was done to control for reward recency at the time of choice. However, for all other time steps in a trial reward events occurred much more sparsely.
- RL-avgRR-2Alphas: This model is identical to RL-avgRR, but separate learning rates were fitted to RL-avgRR$_{part1}$ (α-part1) and RL-avgRR$_{part2}$ (α-part2). Note that, as in RL-avgRR, there was an additional learning rate for the last PE* of RL-avgRR$_{part2}$ (i.e. PE*(LSD-1)) but not for the last PE of RL-avgRR$_{part1}$. Note also that the parameter weights for the two alphas were very similar (median weights of 0.173 and 0.185) explaining why the use of separate learning rates did not further improve model fit.
- RL-avgRR-valuepart1: This model is identical to RL-avgRR, but the value estimate of RL-avgRRpart1 at the time of choice (value(LSD)) is also included in the decision variable of the model, weighted by an additional free parameter *weight-valuepart1* (Decision variable = PE$_{expected}$(LSD) + *weight-valuepart1*×value(LSD)). The rationale for the model is related to one feature of RL-avgRR that might seem counterintuitive — that the decision variable of RL-avgRR PE$_{expected}$, reflects an expected prediction error, and that it does not include an additional non-contextual estimate of the reward environment. Adding such a non-contextual estimate can be done in a very simple way by including the simple value estimate from RL-avgRR$_{part1}$ at the time of choice in the decision variable, which is exactly what was done in this modified model. However, in our experiment this addition to the model did not improve the model fit further in terms of BIC, although a slight decrease of negative log likelihood in the model fitting was observed. This demonstrates that in the context of our study RL-avgRR's PE$_{expected}$ contains sufficient information to reflect both instantaneous reward and reward rate trend. However, to enable the model to be sensitive to absolute reward levels under more stable circumstances, it could include the additional simple value estimate (see Supplementary Fig.4).
- RL-avgRR-freestart: This more exploratory model is identical to RL-avgRR, except that it uses an additional free parameter (start- PE$_{expected}$) as starting value of PE$_{expected}$ on every trial. As the prediction errors were calculated at the beginning of a trial in reference to the initial value one could imagine that this value affects the model fit. Thus, we made a variant of RL-avgRR, which finds the optimal starting value for each subject. However, this did not improve the fit.

In fact, it would be surprising if it had improved the fit, as the important aspect of the model was to differentiate between different environments, for instance ones that had initially high reward rates or initially low reward rates. This is further corroborated by the high correlation of the decision variable that is calculated with this model and with the original RL-avgRR (mean correlation of decision variables is 0.9916, see panel c in Supplementary Fig.3).

Supplementary Methods

Imaging data acquisition and preprocessing. Brain data were collected on a 3 Tesla Siemens Verio MRI scanner equipped with a 32 channel head coil. T1-weighted structural images were acquired using TR=3s, TE=4.75ms, TI = 1100ms, 1x1x1mm voxel size, 256x176x224 grid. Functional images were acquired using a Deichmann echo-planar imaging (EPI) sequence [2] with TR=3s, TE=30 ms, 3x3x3mm voxel size, 87° flip angle, 15° slice angle and z-shimming to reduce signal distortions and dropout in medial orbitofrontal brain areas.

Brain data were analyzed using FMRIB's Software Library (FSL) [3]. FMRI data preprocessing for univariate analyses comprised spatial (Gaussian with full-width half maximum of 5 mm) and temporal filtering (3 dB cut-off at 100 s), motion correction using FSL's MCFLIRT and manual removal of noise components after visual inspection using FSL MELODIC. In a two-step procedure, preprocessed images were nonlinearly registered to Montreal Neurological Institute (MNI) space via subjects' structural images.

FMRI whole-brain analyses. FSL FEAT [3] was used for first level analyses. The fMRI time course was pre-whitened with FSL FILM to account for temporal autocorrelations. Motion regressors derived from MCFLIRT were included as nuisance regressors. Temporal derivatives of the regressors of interest were included and the model was temporally filtered before it was applied to the data. Group results on the second level were calculated with FSL FLAME 1 using outlier de-weighting and a cluster-forming threshold of $z>2.3$ and $p<0.05$. The only exception from this is presented in Fig.6b for illustration.

We ran three fMRI designs; one based on the objective history of reward rates experienced at time of choice and the one derived from our RL-avgRR model. Both designs modeled button presses as stick functions of no interest. The first (***fMRI GLM1;*** Fig.4a) contained a constant regressor time-locked to the leave-stay decision. This constant captured the duration of the last reward event (800ms) and the subsequent choice phase (2,000ms). We used lastRR, avgRR and the logarithm of the reaction times (logRT) as parametric modulators of the constant regressor. Based on the parameter estimates of lastRR and avgRR, we created the contrast lastRR-avgRR. We used this design to identify brain regions sensitive to the reward rate trend. ***fMRI GLM2*** (Fig.5a,b) was built identically, except for the parametric modulators of the LSD regressor. They comprised $PE_{expected}$(LSD-1) (abbreviated in the main text as just "$PE_{expected}$"), which is the expected prediction error, calculated with RL-avgRR, at the last time step before LSD. The only difference to the DV, i.e. to the expected prediction error at the time of the LSD ($PE_{expected}$(LSD); see equation 11) is that it had not yet been updated by the PE* of the last time step. In addition, it contained a binary choice regressor coding trials in which subjects decided to stay in a patch as "1" and trials in which they decided to leave as "-1", and logRT. Correlations between

$PE_{expected}$(LSD-1) and choice are shown in supplementary Figure 7b for all subjects. ***fMRI GLM3*** (Fig.6) was also identical to the previous GLMs with the exception of the parametric regressors used. Again, we used $PE_{expected}$(LSD-1) and logRT. In addition we included the value estimate at the time of the last reward delivery and the magnitude of the last reward: value(LSD-1) and outcome(LSD-1). We calculated a standard prediction error on the contrast level by subtracting the value from the outcome regressor (Fig.6b). Note that for this GLM, RL-avgRR was fitted on the whole group instead of single subjects individually. Although individual fits resulted in a set of regressors that were sufficiently decorrelated for the majority of subjects, this was not the case for all of them. In these and all subsequent region of interest (ROI) analyses, parametric and binary regressors were normalized. Additional fMRI analyses reported in the supplements are described in detail in the supplemental figure legends.

FMRI ROI analyses. ROIs had a radius of three voxels and were centered on peak voxels of significant clusters from fMRI GLM1 and fMRI GLM2 (Supplementary Table 2). To guarantee statistical independence of ROI selection and ROI analyses, we used a leave-one-out procedure to identify ROI peak voxels for the analyses of main effects for areas identified in fMRI GLM 1 (Fig.4b,e,5c,6a; Supplementary Fig.5b,6a,b,e,7c). For this, we conducted group level analyses, leaving one subject out at a time. From the results of the remaining 19 subjects, we extracted local maxima of the relevant clusters and centered the ROIs for the left out subject on the local maxima. We repeated this for all 20 subjects. Therefore, the ROI selection was statistically independent from the data of the subject that was subsequently analyzed in the ROI. For fMRI GLM2, no leave-one-out procedure was necessary because we did not test signals for significance in those ROIs that were related to the defining contrast. Note that correlation analyses were still conducted on the ROI centered on the peak of the whole group because those analyses are not affected by this potential problem.

For ROI time course analyses, we extracted the pre-processed BOLD time courses from each ROI, averaged over all voxels of each volume. The time courses were normalized (per session, as for subsequent analyses), oversampled by a factor of 20 (using cubic spline interpolation, as for subsequent analyses) and, in a trial wise manner, aligned at the time of LSDs. We then applied a GLM to each time point and computed one beta weight per time point, which resulted in a time course of beta weights for each regressor. We used two features of these beta weight time courses within an analysis window of four to thirteen seconds after LSD onset to investigate their relation to individual variance in behavior. First, to compute the slope of the beta weight time course signal, we identified the subject specific absolute (positive or negative) peak of the beta weight time course within the analysis window and fitted a straight line on the time course from the time of LSD onset to the time of the absolute peak. Second, we averaged the time courses across subjects and identified the absolute beta weight group peak within the analysis window. We then took each

subject's beta weight at that time point and examined its relation to behavior. FMRI GLM1 was calculated as a ROI time course analysis and resulting neural signals were correlated with lastRR and avgRR beta weights from behavioral GLM2 (Fig.2b). fMRI GLM2 and fMRI GLM3, too, was calculated as a time course analysis and group peak signals were identified for significance testing (see below). Lastly, **f*MRI GLM4*** was analogous to behavioral GLM1; it employed the reward rates in five discrete time bins relative to the leave-stay decision as well as logRT as parametric regressors (Fig.4e,f). Group peak signals from this time course analysis are shown in Figure 4e and supplementary Figure 6a,b,e. Correlations with behavior were calculated using behavioural GLM1.

We performed significance testing on time course analyses using a leave-one-out procedure on the group peak signal (Fig.5c,6a, Supplementary Fig.7c) to avoid potential temporal selection biases. For every subject, we calculated the time course of the group mean beta weights of the relevant regressor based on the remaining 19 subjects. We then identified the (positive or negative) group peak of the regressor of interest within the analysis window of four to thirteen seconds from LSD onset. Then, we took the beta weight of the remaining subject at the time of the group peak. We repeated this for all subjects. Therefore, the resulting 20 "peak" beta weights were selected independently from the time course of the subject analyzed. We assessed significance using t-tests on the resulting beta weights.

Supplementary References

1. Kolling, N., Behrens, T., Wittmann, M.K. & Rushworth, M. Multiple signals in anterior cingulate cortex. *Curr. Opin. Neurobiol.* **37**, 36-43 (2016).
2. Deichmann, R., Gottfried, J.A., Hutton, C. & Turner, R. Optimized EPI for fMRI studies of the orbitofrontal cortex. *NeuroImage* **19**, 430-441 (2003).
3. Smith, S.M., *et al.* Advances in functional and structural MR image analysis and implementation as FSL. *NeuroImage* **23 Suppl 1**, S208-219 (2004).
